# Glycated hemoglobin thresholds for detecting 2-hour plasma glucose-defined abnormal glucose metabolism in Vietnamese women with polyendocrine metabolic ovarian syndrome

**DOI:** 10.3389/fendo.2026.1968625

**Published:** 2026-09-30

**Authors:** Nam T. Nguyen, Phuc D. Truong, Hien T. A. Nguyen, Toan D. Pham, Minh H. N. Nguyen, Duy L. Nguyen, Vu N. A. Ho, Tuong M. Ho, Lan N. Vuong

**Affiliations:** 1 IVFMD, My Duc Hospital, Ho Chi Minh City, Vietnam; 2 HOPE Research Center, My Duc Hospital, Ho Chi Minh City, Vietnam; 3 Endocrine Clinic, My Duc Hospital, Ho Chi Minh City, Vietnam; 4 Department of Obstetrics and Gynaecology, University of Medicine and Pharmacy at Ho Chi Minh City, Ho Chi Minh City, Vietnam

**Keywords:** fasting plasma glucose, glycated hemoglobin, infertility, oral glucose tolerance test, polyendocrine metabolic ovarian syndrome, Vietnamese

## Abstract

**Objective:**

To determine glycated hemoglobin (HbA1c) thresholds for detecting 2-hour plasma glucose (2hPG)-defined post-load dysglycemia and diabetes-range hyperglycemia, and to assess whether adding fasting plasma glucose (FPG) improves discrimination in Vietnamese women with polyendocrine metabolic ovarian syndrome (PMOS).

**Methods:**

We performed a secondary diagnostic-accuracy analysis of a prospective cohort of 1,149 women with PMOS seeking fertility care. FPG was measured immediately before glucose administration at the OGTT visit after an overnight fast of at least 8 hours. The reference standard was 2hPG during a 75-g oral glucose tolerance test (OGTT): post-load dysglycemia was defined as 2hPG ≥140 mg/dL and diabetes-range 2hPG hyperglycemia was defined as a single 2hPG ≥200 mg/dL. Discrimination was assessed using receiver operating characteristic curves, area under the curve (AUC), Youden-optimal thresholds, paired DeLong tests, and bootstrap validation.

**Results:**

Of the 1,149 women, 342 (29.8%) had 2hPG-defined post-load dysglycemia, including 61 (5.3% of the total cohort) with 2hPG-defined diabetes-range hyperglycemia. HbA1c outperformed FPG for both outcomes (AUC, 0.711 vs 0.634 and 0.799 vs 0.719, respectively). The Youden-optimal HbA1c threshold was 5.58% (37 mmol/mol) for 2hPG-defined post-load dysglycemia (sensitivity, 53.8%; specificity, 80.5%) and 5.62% (38 mmol/mol) for 2hPG-defined diabetes-range hyperglycemia (sensitivity, 68.9%; specificity, 80.0%). The Youden-derived cutoffs are exploratory statistical reference points. For the diabetes-range endpoint, an exploratory HbA1c cutoff of 5.3% (34 mmol/mol) achieved a point-estimate sensitivity of 93.4% and specificity of 34.5%, but would lead to OGTT in 670 per 1,000 women screened. Adding FPG produced small numerical increases in AUC, but there was no statistically significant evidence of improved discrimination over HbA1c alone; bootstrap validation of the combined models showed minimal optimism.

**Conclusion:**

Among Vietnamese women with PMOS seeking fertility care, HbA1c may help prioritize confirmatory OGTT, but it cannot rule out 2hPG-defined post-load dysglycemia or replace OGTT for definitive glycemic classification. The Youden-derived thresholds should be considered exploratory, and external validation is required before clinical use.

## 1 Introduction

Polyendocrine metabolic ovarian syndrome (PMOS), formerly known as polycystic ovary syndrome (PCOS), is a common endocrine-metabolic disorder affecting approximately 10–13% of women of reproductive age and is characterized by heterogeneous reproductive, dermatological, psychological, and metabolic manifestations (1, 2). Beyond ovulatory dysfunction and hyperandrogenism, insulin resistance is a central feature of PMOS and contributes to a higher lifetime risk of abnormal glucose metabolism, including prediabetes and type 2 diabetes mellitus (3, 4). This metabolic vulnerability may occur independently of body mass index (BMI), underscoring the need for active glycemic assessment even in women who are not obese (4–6).

Metabolic risk assessment is particularly relevant in Vietnamese women with PMOS seeking fertility care because abnormal glucose metabolism may occur despite relatively low BMI. A previous prospective cohort of 1,208 infertile Vietnamese women diagnosed with PMOS found that abnormal glucose metabolism defined using the study’s composite glycemic criteria was common, affecting 614 women (50.8%); of these, 494 had prediabetes and 120 had type 2 diabetes (7). This burden was observed in a population of infertile women with relatively low BMI, suggesting that conventional anthropometric thresholds alone may be insufficient to identify metabolic risk in this clinical setting. These data are clinically relevant for fertility care, where unrecognized abnormal glucose metabolism may affect preconception counseling, pregnancy planning, and long-term cardiometabolic prevention.

The 2026 American Diabetes Association (ADA) Standards of Care define prediabetes and diabetes using fasting plasma glucose (FPG), 2-hour plasma glucose (2hPG) during a 75-g oral glucose tolerance test (OGTT), and glycated hemoglobin (HbA1c); diabetes can also be diagnosed using random plasma glucose in individuals with classic hyperglycemic symptoms or hyperglycemic crisis (8). In the absence of unequivocal hyperglycemia, a diabetes diagnosis requires confirmatory testing (8). In PMOS, the 2023 International Evidence-based Guideline recommends an OGTT as the most accurate test for assessing glycemic status, regardless of BMI (1). However, the OGTT requires fasting, multiple blood samples, and a two-hour testing period, which may reduce patient uptake and limit routine implementation in busy fertility clinics and resource-constrained settings. FPG is simpler but may miss a substantial proportion of women with post-load hyperglycemia, particularly those with isolated 2hPG abnormalities (9, 10). HbA1c is operationally attractive because it does not require fasting, has lower short-term biological variability, and reflects average glycemia over the preceding 2–3 months (8).

Nevertheless, the performance of HbA1c in women with PMOS remains uncertain. The conventional ADA HbA1c thresholds of 5.7–6.4% (39 to 46 mmol/mol) for prediabetes and ≥6.5% (48 mmol/mol) for diabetes were developed for general populations and may not optimally capture early abnormal glucose metabolism in younger Asian women with relatively low BMI and PMOS (8). Previous studies in PMOS have shown only modest agreement between HbA1c and OGTT for detecting prediabetes or diabetes, raising concerns that HbA1c used at conventional cutoff values may under-detect abnormal glucose metabolism in this population (11–13). A recent systematic review also emphasized the limited diagnostic accuracy of HbA1c and FPG compared with OGTT among women with PMOS, supporting the need for context-specific screening strategies rather than direct extrapolation from criteria used in the general population (14).

Population-specific evidence from Asian cohorts further supports reassessing HbA1c thresholds. In Asian Indian populations, an HbA1c cutoff of around 5.6% (38 mmol/mol) has been reported to better identify impaired fasting glucose and impaired glucose tolerance than the conventional 5.7% (39 mmol/mol) threshold (15). Multi-ethnic Singaporean data also showed variability in HbA1c performance across Asian ethnic groups, reinforcing the importance of ethnicity- and population-specific interpretation (16). Given the distinctive metabolic profile of Asian women with PMOS, including relatively low BMI but a high prevalence of abnormal glucose metabolism, a locally calibrated HbA1c threshold may improve identification of women who should be prioritized for confirmatory OGTT.

Accordingly, this study aimed to identify HbA1c thresholds for detecting 2hPG-defined post-load dysglycemia and the nested endpoint of diabetes-range 2hPG hyperglycemia in Vietnamese women with PMOS seeking fertility care, and to assess whether adding FPG improved diagnostic discrimination. The broader clinical aim was to evaluate whether HbA1c could serve as a pragmatic first-step triage tool for prioritizing confirmatory OGTT when universal OGTT implementation is challenging.

## 2 Materials and methods

### 2.1 Study design

This was a diagnostic-accuracy secondary analysis of a prospective cohort study conducted at a single fertility center in Vietnam between June 2020 and August 2024. The original prospective cohort study was registered at ClinicalTrials.gov (NCT04364087) (7). The protocol for this secondary analysis was prespecified in a separate institutional secondary-analysis protocol, version 1.0, dated 21 October 2025. The protocol was approved by the My Duc Hospital Ethics Review Board on 3 December 2025 (approval number 21/25/DD-BVMD), and the original prospective cohort study was approved by the same board (approval number 08/20/ĐĐ-BVMD; April 24, 2020). The original study was conducted in accordance with the Declaration of Helsinki, and all participants provided written informed consent before enrollment. The statistical analysis plan was incorporated into the approved protocol, which is retained by My Duc Hospital and is available from the corresponding author upon request.

### 2.2 Participants

The prospective cohort study enrolled women aged 18–40 years presenting with infertility who fulfilled the Rotterdam 2003 diagnostic criteria for PCOS, which was the terminology used when that study was designed. In our study, we use PMOS to reflect the 2026 international terminology update (2) while retaining the original Rotterdam diagnostic criteria. Participants were diagnosed as having PCOS when at least two of the following three criteria were present: ovulatory dysfunction (OD), clinical or biochemical hyperandrogenism (HA), and polycystic ovarian morphology (PCOM) (17). PCOS phenotypes were classified according to NIH 2012 criteria (A: HA + OD + PCOM; B: HA + OD; C: HA + PCOM; D: OD + PCOM) (18, 19). Women with thyroid dysfunction, prolactin >30 ng/mL, prior hypothyroidism, Cushing’s syndrome, premature ovarian insufficiency or non-classical congenital adrenal hyperplasia were excluded (7).

### 2.3 Index tests

At the first visit, HbA1c and a plasma glucose measurement obtained after a minimum 4-hour fast were collected. Participants for whom an OGTT remained indicated were instructed to maintain their usual diet for at least 3 days and return within 3 months for a 75-g OGTT after an overnight fast of at least 8 hours. At the OGTT visit, fasting plasma glucose (FPG), which served as the FPG index test in the primary analysis, was measured immediately before glucose administration; 2hPG was measured 2 hours later. The first-visit glucose measurement obtained after the protocol minimum fast of 4 hours does not meet the conventional definition of FPG and was retained only in explicitly labeled sensitivity analyses (**Supplementary Table S9**). No glucose-lowering treatment was initiated or modified by the endocrinology team between the first visit and completion of the OGTT; definitive glycemic classification and referral for treatment occurred only after the complete OGTT results were available. HbA1c was measured using a Tosoh HLC-723GX analyzer (Tosoh Bioscience, Japan), an NGSP- and IFCC-certified system based on the reference method used in the DCCT, with an analytical coefficient of variation (CV) of 1.0%. Plasma glucose was measured on a Beckman Coulter AU480 analyzer (CV 0.9%). Laboratory quality was maintained through routine internal quality control and participation in external quality assessment programs. Participants who had not achieved the ≥4-hour protocol minimum at the first visit were rescheduled for the first-visit glucose measurement (6). Personnel performing the index test did not have access to, and were not involved in, the classification of glycemic status according to the reference standard.

### 2.4 Reference standard

After at least 3 days on a regular diet and overnight fasting for at least 8 hours, participants underwent a 75-g OGTT at a second visit, with venous blood drawn in the fasting state and 2 hours after glucose ingestion. The primary reference standard for this diagnostic-accuracy analysis was based only on 2hPG during OGTT. Post-load dysglycemia was defined as 2hPG ≥140 mg/dL, including impaired glucose tolerance (2hPG 140 to <200 mg/dL). A nested higher-severity endpoint, diabetes-range 2hPG hyperglycemia, was defined as a single 2hPG ≥200 mg/dL. Because HbA1c and FPG were the index tests under evaluation, they were not included in the primary reference-standard definition to avoid incorporation bias and inflation of apparent diagnostic accuracy. As a sensitivity analysis, diagnostic accuracy was assessed against composite glycemic definitions using either first-visit glucose or OGTT-visit FPG as the glucose component (**Supplementary Table S5**). These analyses were considered circular because index-test information contributed to the reference definitions. The diabetes-range endpoint was based on a single 2hPG measurement and was not interpreted as a confirmed clinical diagnosis; consistent with the 2026 ADA Standards, confirmatory testing is required in the absence of unequivocal hyperglycemia (8).

### 2.5 Statistical analysis

Continuous variables are summarized as mean ± standard deviation when distributions were visually symmetric or as median (interquartile range); categorical variables are reported as number (%). Between-group comparisons across 2hPG-defined glycemic categories were performed using one-way analysis of variance (ANOVA) or the Kruskal-Wallis test for continuous variables and the Chi-squared or Fisher exact test for categorical variables, with Holm correction for pairwise *post-hoc* comparisons.

Diagnostic accuracy was assessed using receiver operating characteristic (ROC) curves generated from continuous HbA1c and FPG values for the single-marker analyses and from continuous predicted probabilities for the combined HbA1c + FPG model. Area under the curve (AUC) and 95% confidence interval (CI) values were computed using DeLong’s method (Sun & Xu fast implementation) (20). The Youden index identified the optimal cutoff. At each operating point we report sensitivity, specificity, positive predictive value and negative predictive value with Wilson 95% CI values, and positive and negative likelihood ratios with 95% CI values computed on the log scale. All paired AUC comparisons were conducted in the same complete-case population of 1,149 women using paired DeLong tests. Differences were calculated as the AUC of the first test minus that of the second test and are reported with 95% CIs and exact p values. The combined model is logit(p) = b0 + b1 x HbA1c + b2 x FPG, with HbA1c in NGSP percent and FPG in mmol/L, both continuous and untransformed, fitted by unpenalized maximum likelihood with the lbfgs solver (**Supplementary Table S10**). Departure from linearity was tested by comparing a restricted cubic spline with three knots at the 10th, 50th and 90th percentiles against the linear term by likelihood ratio (**Supplementary Table S11**). Sample size was justified by the precision of the sensitivity estimate rather than by a hypothesis test: with 342 reference-positive women, the sensitivity at the selected cutoff is estimated with a Wilson half-width of 5.3 percentage points, and with 61 women at the 2hPG-defined diabetes-range endpoint, the half-width is 11.3 points (**Supplementary Table S16**) (21).

The combined HbA1c + FPG model was internally validated using the Harrell bootstrap optimism procedure with 500 resamples, yielding apparent and optimism-corrected AUC, calibration slope and intercept, and Brier score (22). Calibration was further assessed graphically with a flexible Locally Weighted Scatterplot Smoothing calibration plot and decile means (22). The cutoff-selection procedure was validated separately over 1,000 resamples: the Youden-optimal HbA1c cutoff was reselected in each bootstrap sample and applied to the observations left out of it, giving the distribution of selected cutoffs and optimism-corrected sensitivity and specificity (**Supplementary Table S12**). An exploratory point-estimate sensitivity target of 90% for the diabetes-range endpoint was specified before selecting among the candidate operating points for clinical interpretation, and the absolute consequences of each candidate cutoff are reported per 1,000 women screened (**Supplementary Table S13**). Decision-curve analysis was performed as a testing strategy, in which a positive result leads to an OGTT rather than to treatment, across testing-threshold probabilities of 0.02 to 0.20, and is also reported as the net reduction in OGTTs per 1,000 women (**Supplementary Table S14**), to compare the net benefit of HbA1c only, FPG only, the combined model, and the “OGTT-for-all”/”OGTT-for-none” reference strategies (23). Reporting followed Standards for Reporting of Diagnostic Accuracy Studies (STARD) 2015.

An OGTT was not performed in women whose first-visit HbA1c and/or ≥4-hour glucose measurement met the original study’s protocol-defined diabetes-range exclusion criteria; therefore, the reference standard was not applied to these participants and the design is subject to partial verification bias. Its effect was bounded by classifying the unverified participants first as reference-positive and then as reference-negative (**Supplementary Table S15**). We also calculated AUCs after excluding participants with a record of post-classification management with metformin, insulin, or a glucagon-like peptide-1 receptor agonist (**Supplementary Table S6**). Because these variables recorded management assigned after complete glycemic classification rather than medication use at enrollment, this exclusion conditions on a post-classification variable strongly associated with glycemic status and was interpreted as descriptive only, not as a robustness analysis. The distribution of treatment records by 2hPG category is reported in **Supplementary Table S8**. Exploratory subgroup AUCs for the combined model for 2hPG-defined post-load dysglycemia were estimated by BMI (<23 vs ≥23 kg/m²), PMOS phenotype (A–D), and age (<25, 25–30, 31–35, and >35 years); these analyses were descriptive, and no formal interaction tests were performed.

Analyses were performed in Python 3.14.6 with pandas 2.3.3, numpy 2.4.4, statsmodels 0.14.6, scikit-learn 1.8.0, scipy 1.17.1 and matplotlib 3.10.9. A two-sided p value <0.05 was considered statistically significant.

## 3 Results

### 3.1 Study population

Of the 1,208 women enrolled in the original cohort, eight with diabetes diagnosed before the study assessment were excluded. A further 51 women met the original study’s protocol-defined diabetes-range exclusion criteria on first-visit HbA1c and/or the ≥4-hour glucose measurement and did not undergo OGTT. The diagnostic-accuracy cohort therefore comprised 1,149 women with HbA1c, OGTT-visit FPG, and 2hPG data. The OGTT was completed within 1 month by 1,010 women (87.9%), after >1 to 2 months by 48 (4.2%), and after >2 to 3 months by 91 (7.9%); all participants completed the OGTT within 3 months of the first visit. Participant flow and analysis denominators are summarized in **Supplementary Table S1**, and baseline characteristics by 2hPG category in **Supplementary Table S7**. Baseline demographic, anthropometric, and clinical characteristics of the analysis cohort have been reported in detail previously (7). Briefly, using the previously reported composite glycemic classification based on first-visit glucose, women with abnormal glucose metabolism were older and had a higher BMI, larger waist circumference, and a greater prevalence of central obesity than women with normal glucose metabolism. Despite this metabolic gradient, women with abnormal glucose metabolism had a mean BMI of approximately 24.2 kg/m². Metabolic indices, including HbA1c, FPG, 2hPG, and Homeostatic Model Assessment of Insulin Resistance (HOMA-IR) increased across glycemic categories. For the analysis, women were classified according to the 2hPG result during OGTT: 2hPG-defined diabetes-range hyperglycemia and 2hPG-defined post-load dysglycemia. Overall, 807 women (70.2%) had normal 2hPG tolerance, 281 (24.5%) had impaired glucose tolerance, and 61 (5.3%) had 2hPG-defined diabetes-range hyperglycemia. Thus, 342 women (29.8%) had 2hPG-defined post-load dysglycemia.

### 3.2 Diagnostic performance of different models

Diagnostic indices for HbA1c, FPG, and the combined HbA1c + FPG combination at selected operating points are presented in **Table 1**, with complete operating-point estimates provided in **Supplementary Table S2**; corresponding ROC curves are shown in **Figure 1**.

**Table 1:** Diagnostic performance of glycated hemoglobin (HbA1c), fasting plasma glucose (FPG), and the combined HbA1c + FPG model for 2-hour plasma glucose (2hPG)-defined post-load dysglycemia and 2hPG-defined diabetes-range hyperglycemia (n=1,149).

Endpoint	Test	Cutoff type	AUC (95% CI)	Sensitivity (95% CI)	Specificity (95% CI)	PPV	NPV	Events
Post-load dysglycemia (2hPG ≥140 mg/dL)	HbA1c	ADA threshold/5.7% (39 mmol/mol)	0.711 (0.677-0.745)	0.380 (0.330-0.433)	0.887 (0.864-0.907)	0.588 (0.522-0.651)	0.772 (0.743-0.797)	342
		ADA threshold/6.5% (48 mmol/mol)	0.711 (0.677-0.745)	0.000 (0.000-0.011)	1.000 (0.995-1.000)	NE	0.702 (0.675-0.728)	342
		Youden/5.58% (37 mmol/mol)	0.711 (0.677-0.745)	0.538 (0.485-0.590)	0.805 (0.777-0.831)	0.540 (0.487-0.592)	0.804 (0.776-0.830)	342
		Sensitivity >=90%/5.13% (33 mmol/mol)	0.711 (0.677-0.745)	0.901 (0.864-0.928)	0.202 (0.176-0.231)	0.324 (0.295-0.354)	0.827 (0.769-0.874)	342
	FPG	ADA threshold/5.6 mmol/L (100 mg/dL)	0.634 (0.597-0.670)	0.292 (0.247-0.343)	0.895 (0.872-0.914)	0.541 (0.469-0.611)	0.749 (0.721-0.775)	342
		ADA threshold/7 mmol/L (126 mg/dL)	0.634 (0.597-0.670)	0.006 (0.002-0.021)	1.000 (0.995-1.000)	1.000 (0.342-1.000)	0.704 (0.677-0.729)	342
		Youden/5.28 mmol/L (95 mg/dL)	0.634 (0.597-0.670)	0.523 (0.470-0.576)	0.699 (0.666-0.730)	0.424 (0.378-0.472)	0.776 (0.744-0.805)	342
		Sensitivity >=90%/4.7 mmol/L (85 mg/dL)	0.634 (0.597-0.670)	0.906 (0.871-0.933)	0.139 (0.117-0.164)	0.308 (0.281-0.338)	0.778 (0.703-0.838)	342
	HbA1c + FPG	Youden/predicted probability ≥0.313	0.721 (0.687-0.755)	0.623 (0.570-0.673)	0.736 (0.705-0.765)	0.500 (0.453-0.547)	0.822 (0.792-0.848)	342
		Specificity >=0.90/predicted probability ≥0.449	0.721 (0.687-0.755)	0.365 (0.316-0.418)	0.901 (0.878-0.920)	0.610 (0.542-0.674)	0.770 (0.742-0.796)	342
Diabetes-range hyperglycemia (single 2hPG result ≥200 mg/dL)	HbA1c	ADA threshold/5.7% (39 mmol/mol)	0.799 (0.739-0.858)	0.590 (0.465-0.705)	0.830 (0.806-0.851)	0.163 (0.120-0.217)	0.973 (0.961-0.982)	61
		ADA threshold/6.5% (48 mmol/mol)	0.799 (0.739-0.858)	0.000 (0.000-0.059)	1.000 (0.996-1.000)	NE	0.947 (0.932-0.958)	61
		Youden/5.62% (38 mmol/mol)	0.799 (0.739-0.858)	0.689 (0.564-0.791)	0.800 (0.775-0.822)	0.162 (0.122-0.211)	0.979 (0.967-0.986)	61
		Sensitivity >=90%/5.3% (34 mmol/mol)	0.799 (0.739-0.858)	0.934 (0.843-0.974)	0.345 (0.317-0.373)	0.074 (0.058-0.095)	0.989 (0.973-0.996)	61
	FPG	ADA threshold/5.6 mmol/L (100 mg/dL)	0.719 (0.650-0.788)	0.443 (0.325-0.567)	0.855 (0.833-0.874)	0.146 (0.102-0.204)	0.965 (0.951-0.975)	61
		ADA threshold/7 mmol/L (126 mg/dL)	0.719 (0.650-0.788)	0.033 (0.009-0.112)	1.000 (0.996-1.000)	1.000 (0.342-1.000)	0.949 (0.934-0.960)	61
		Youden/5.5 mmol/L (99 mg/dL)	0.719 (0.650-0.788)	0.541 (0.417-0.660)	0.792 (0.767-0.815)	0.127 (0.092-0.174)	0.969 (0.955-0.978)	61
		Sensitivity >=90%/4.9 mmol/L (88 mg/dL)	0.719 (0.650-0.788)	0.902 (0.802-0.954)	0.261 (0.236-0.288)	0.064 (0.050-0.082)	0.979 (0.956-0.990)	61
	HbA1c + FPG	Youden/predicted probability ≥0.047	0.821 (0.764-0.877)	0.787 (0.669-0.871)	0.747 (0.721-0.772)	0.149 (0.114-0.192)	0.984 (0.973-0.991)	61
		Specificity >=0.90/predicted probability ≥0.101	0.821 (0.764-0.877)	0.541 (0.417-0.660)	0.902 (0.883-0.918)	0.236 (0.173-0.312)	0.972 (0.960-0.981)	61

Values are diagnostic performance estimates (95% confidence interval) where applicable. Sensitivity, specificity, PPV and NPV intervals use the Wilson method; likelihood-ratio intervals in the **Supplementary Tables** are computed on the log scale. When a zero cell prevented estimation of a finite likelihood ratio and its log-scale confidence interval, the affected likelihood ratio was reported as NE. Prespecified cutoffs refer to probability thresholds for the combined HbA1c + FPG model. ADA, American Diabetes Association; AUC, area under the curve; CI, confidence interval; FPG, fasting plasma glucose; 2hPG, 2-hour plasma glucose; HbA1c, glycated hemoglobin; NPV, negative predictive value; PPV, positive predictive value.

**Figure 1:**
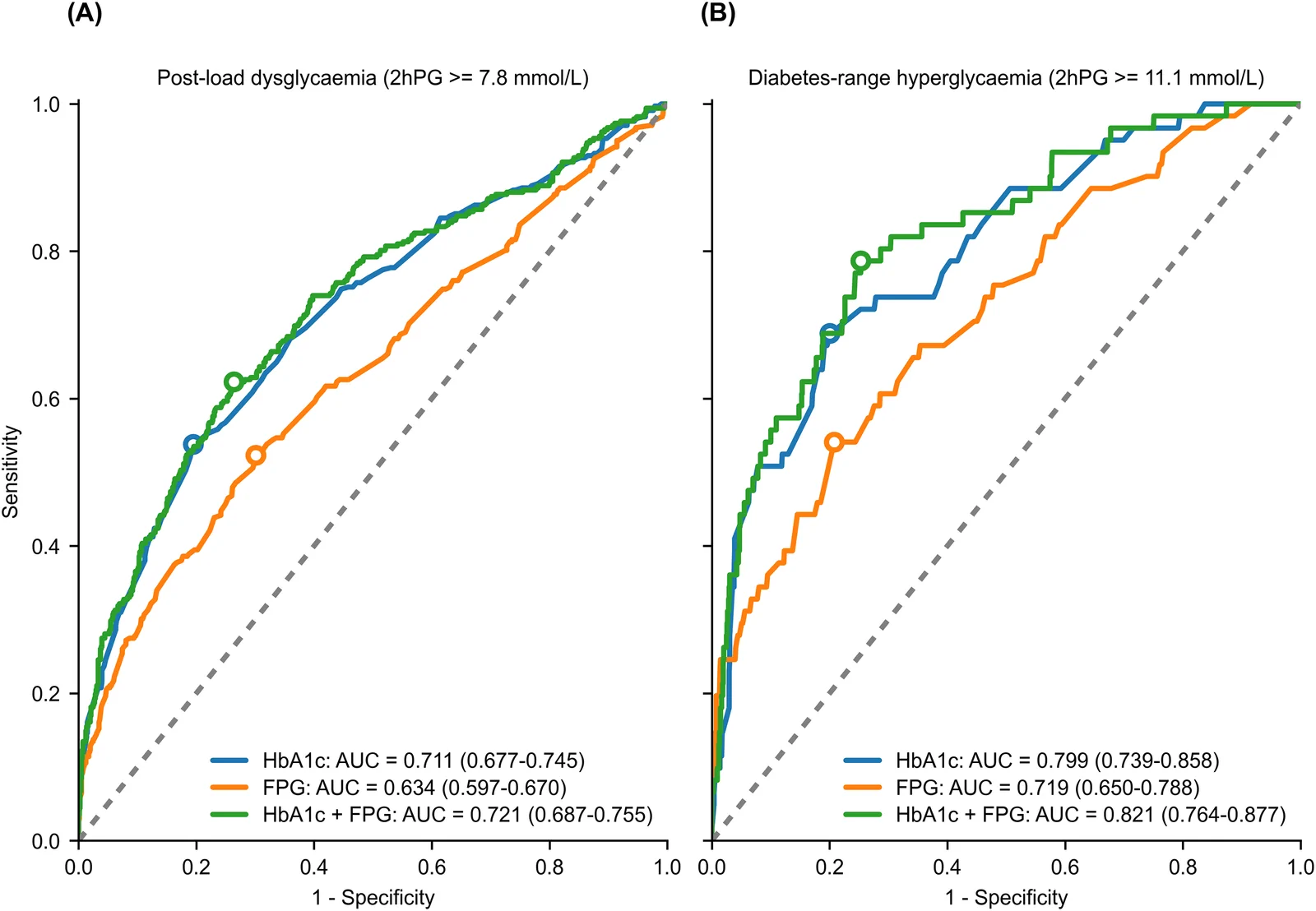
Receiver operating characteristic curves for glycated hemoglobin (HbA1c), fasting plasma glucose (FPG), and the combined HbA1c + FPG model for detecting 2-hour plasma glucose (2hPG)-defined post-load dysglycemia **(A)** and 2hPG-defined diabetes-range hyperglycemia **(B)** on oral glucose tolerance test. Open circles mark Youden-optimal operating points; AUC, area under the curve; CI, confidence interval.

For 2hPG-defined post-load dysglycemia, the AUCs were 0.711 (95% CI 0.677 to 0.745) for HbA1c, 0.634 (0.597 to 0.670) for FPG, and 0.721 (0.687 to 0.755) for the combined model. HbA1c had a higher AUC than FPG, with a paired difference of +0.077 (95% CI + 0.035 to +0.120; p<0.001). The combined model yielded a numerically higher AUC than HbA1c alone, with a paired difference calculated as HbA1c minus the combined model of −0.010 (−0.023 to +0.002; p=0.105).

For diabetes-range 2hPG hyperglycemia, the corresponding AUCs were 0.799 (95% CI 0.739 to 0.858) for HbA1c, 0.719 (0.650 to 0.788) for FPG, and 0.821 (0.764 to 0.877) for the combined model. The paired difference between HbA1c and FPG was +0.080 (95% CI + 0.005 to +0.155; p=0.037), whereas the difference between HbA1c and the combined model was −0.022 (−0.049 to +0.005; p=0.105). Thus, adding FPG produced small numerical increases in AUC, but there was no statistically significant evidence of improved discrimination over HbA1c alone (**Supplementary Table S3**).

The Youden-optimal HbA1c cutoff for post-load dysglycemia was 5.58% (37 mmol/mol), with a sensitivity of 53.8% and specificity of 80.5%. For diabetes-range 2hPG hyperglycemia, the corresponding cutoff was 5.62% (38 mmol/mol), with a sensitivity of 68.9% and specificity of 80.0%.

For 2hPG-defined post-load dysglycemia, the highest HbA1c cutoff with an observed sensitivity of at least 90% was 5.13% (33 mmol/mol), yielding a sensitivity of 90.1% (95% CI 86.4–92.8), specificity of 20.2% (17.6–23.1), and negative predictive value of 82.7% (76.9–87.4). For diabetes-range 2hPG hyperglycemia, the highest evaluated cutoff meeting the exploratory point-estimate sensitivity target of at least 90% was 5.3% (34 mmol/mol), with a sensitivity of 93.4% (84.3–97.4), specificity of 34.5% (31.7–37.3), and negative predictive value of 98.9% (97.3–99.6). At the prevalence observed in this cohort, use of the 5.3% cutoff would result in 670 OGTTs per 1,000 women screened (**Table 1**; **Supplementary Table S13**).

### 3.3 Risk-group composition under prespecified probability cutoffs

Risk-group composition under the prespecified probability cutoff values is shown in **Figure 2**. For 2hPG-defined post-load dysglycemia, the observed prevalence increased from 21.6% in the low-risk group (n=888) to 51.4% in the moderate-risk group (n=218) and 88.4% in the high-risk group (n=43). The 2hPG-defined diabetes-range hyperglycemia model showed a similar gradient, from 2.8% (n=1,007) to 18.9% (n=111) and 38.7% (n=31).

**Figure 2:**
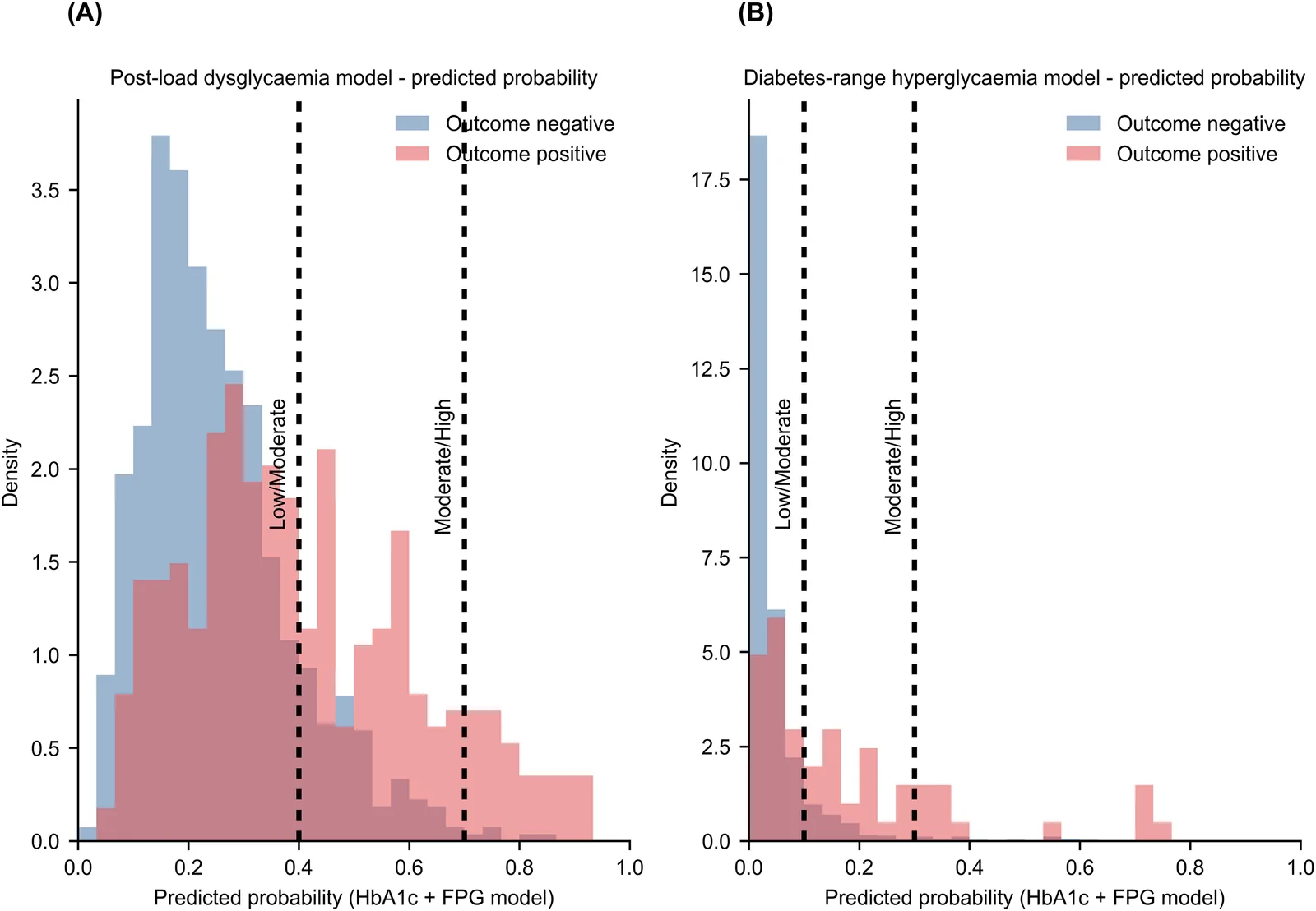
Distribution of predicted probabilities from the combined glycated hemoglobin + fasting plasma glucose model for outcome-positive and outcome-negative women, with prespecified risk-group cutoffs annotated. **(a)** 2-hour plasma glucose (2hPG)-defined post-load dysglycemia; **(b)** 2hPG-defined diabetes-range hyperglycemia. FPG, fasting plasma glucose; HbA1c, glycated hemoglobin.

### 3.4 Internal validation, calibration and decision-curve analysis

After 500 bootstrap resamples, the combined HbA1c + FPG model showed minimal optimism, with apparent and optimism-corrected AUCs differing by <0.01 for both endpoints; calibration slopes were close to 1 and intercepts were close to 0 (**Table 2**; **Supplementary Table S4**). Calibration plots are shown in **Supplementary Figures S1** and **S2**. Across 1,000 bootstrap resamples, the median selected cutoff was 5.56% (2.5th–97.5th percentile, 5.41%–5.58%; 81.6% of resamples within 5.5%–5.7%) for 2hPG-defined post-load dysglycemia and 5.62% (5.56%–5.85%; 89.6% within 5.5%–5.7%) for diabetes-range 2hPG hyperglycemia. The corresponding optimism-corrected sensitivities were 0.524 and 0.628, and the optimism-corrected specificities were 0.799 and 0.797, respectively (**Supplementary Table S12**). Decision-curve analysis (**Figure 3**) showed that the clinical value of HbA1c depended on the endpoint and testing-threshold probability. For 2hPG-defined post-load dysglycemia, the equivalent net reduction in OGTTs relative to OGTT for everyone was 0 to 7 per 1,000 women at p_t=0.02–0.05, was negative (−60 to −1) at p_t=0.06–0.16, and ranged from 14 to 78 per 1,000 at p_t=0.17–0.20, indicating no consistent or clinically meaningful advantage across most of the evaluated range. For 2hPG-defined diabetes-range hyperglycemia, HbA1c had greater net benefit than both OGTT for everyone and OGTT for no one throughout p_t=0.02–0.20. The corresponding equivalent net reductions relative to OGTT for everyone were 87, 498, and 775 per 1,000 women at p_t=0.02, 0.06, and 0.20, respectively; the net benefit of OGTT for everyone was negative from p_t=0.06 onward (**Supplementary Table S14**). The combined model did not consistently outperform HbA1c alone.

**Table 2:** Internal validation of the combined glycated hemoglobin + fasting plasma glucose model.

Model/endpoint	n	Events	AUC apparent (95% CI)	AUC optimism-corrected	Brier apparent	Brier optimism-corrected	Calibration slope (corrected)	Calibration intercept (corrected)	Bootstrap (n)
(HbA1c + FPG)/Post-load dysglycemia	1149	342	0.721	0.720	0.177	0.178	0.996	-0.000	500
(HbA1c + FPG)/Diabetes-range hyperglycemia	1149	61	0.821	0.816	0.044	0.045	0.981	+0.002	500
HbA1c/Post-load dysglycemia	1149	342	0.711 (0.677-0.745)	—	—	—	—	—	—
FPG/Post-load dysglycemia	1149	342	0.634 (0.597-0.670)	—	—	—	—	—	—
HbA1c/Diabetes-range hyperglycemia	1149	61	0.799 (0.739-0.858)	—	—	—	—	—	—
FPG/Diabetes-range hyperglycemia	1149	61	0.719 (0.650-0.788)	—	—	—	—	—	—

AUC, area under the curve; CI, confidence interval; FPG, fasting plasma glucose; HbA1c, glycated hemoglobin.

**Figure 3:**
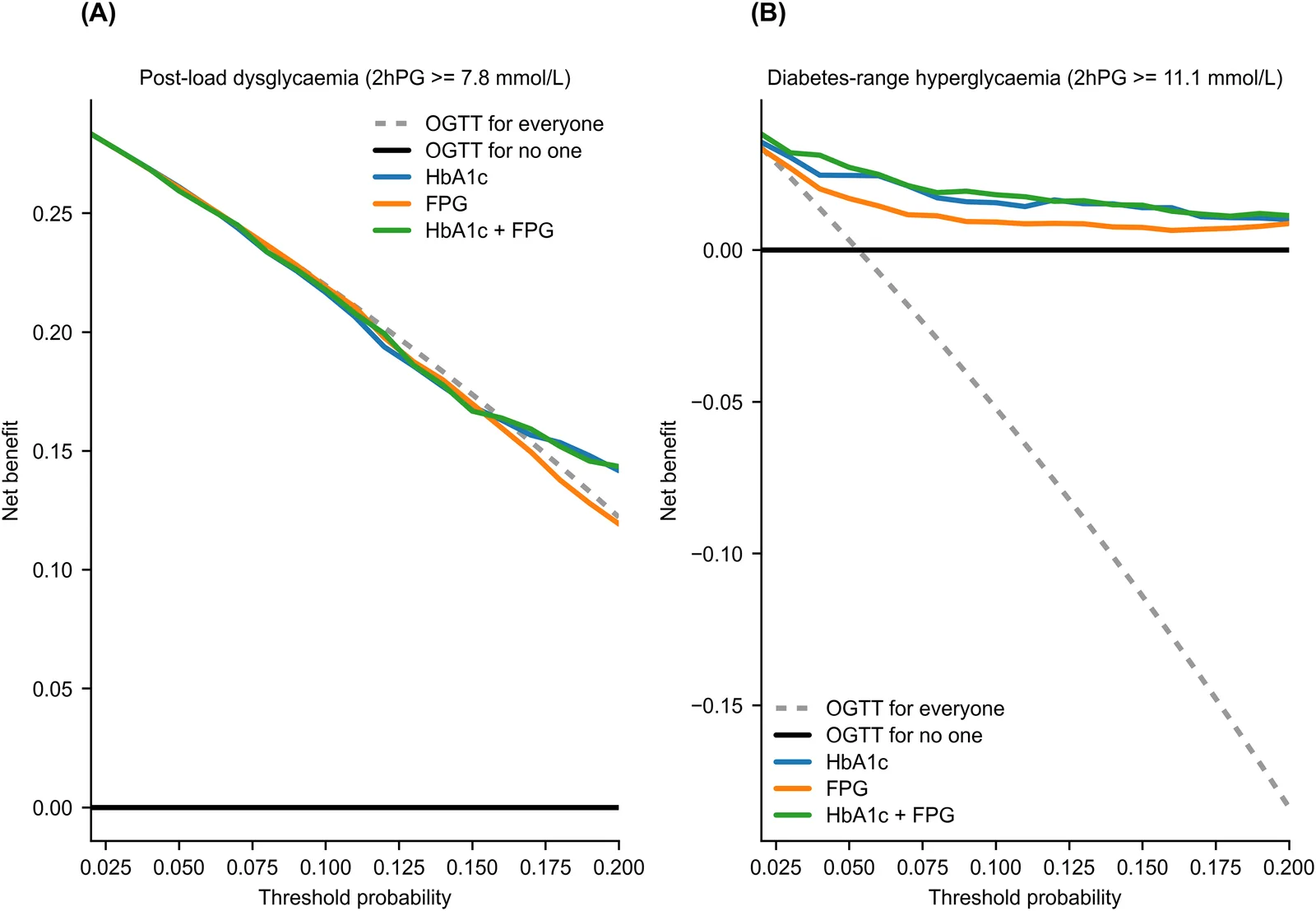
Decision-curve analysis interpreted as a testing strategy, in which an estimated outcome probability at or above the testing-threshold probability leads to a 75-g oral glucose tolerance test (OGTT) rather than treatment. Net benefit is shown for HbA1c alone, fasting plasma glucose (FPG) alone, and the combined HbA1c + FPG model, with “OGTT for everyone” and “OGTT for no one” as reference strategies, across testing-threshold probabilities of 0.02 to 0.20. **(a)** 2-hour plasma glucose (2hPG)-defined post-load dysglycemia (2hPG ≥7.8 mmol/L); **(b)** 2hPG-defined diabetes-range hyperglycemia (2hPG ≥11.1 mmol/L).

### 3.5 Sensitivity and exploratory analyses

For dysglycemia, both composite definitions yielded higher apparent AUCs for HbA1c and FPG than the 2hPG-only reference standard (**Supplementary Table S5**). After excluding 181 participants recorded as receiving metformin, insulin, or a glucagon-like peptide-1 receptor agonist, 968 remained; the ranking of the AUC point estimates across tests was unchanged, but only 10 diabetes-range 2hPG events remained and the estimates were imprecise (**Supplementary Table S6**). Classifying the 51 unverified participants with index-test results first as reference-negative and then as reference-positive placed the HbA1c AUC between 0.672 and 0.744 for post-load dysglycemia and between 0.767 and 0.870 for diabetes-range 2hPG hyperglycemia; the corresponding complete-case estimates of 0.711 and 0.799 fell within both ranges (**Supplementary Table S15**). Exploratory subgroup AUC estimates for the combined model for 2hPG-defined post-load dysglycemia, including the lower-BMI subgroup (BMI <23 kg/m²) and higher-BMI subgroup (BMI ≥23 kg/m²), are shown in **Supplementary Figure S3**. Confidence intervals overlapped and were wide in several smaller subgroups; without formal interaction tests, no between-subgroup differences were inferred.

## 4 Discussion

In a population of Vietnamese women with PMOS seeking fertility care, who are characterized by a distinctive metabolic pattern (4, 5, 7), our analysis showed that HbA1c was the best-performing single index test evaluated for detecting 2hPG-defined post-load dysglycemia and 2hPG-defined diabetes-range hyperglycemia, although discrimination was modest for 2hPG-defined post-load dysglycemia. The Youden-derived HbA1c thresholds were approximately 5.6% (38 mmol/mol) for both endpoints. FPG showed lower discrimination than HbA1c for both endpoints. Adding FPG to HbA1c produced small numerical increases in AUC, but there was no statistically significant evidence of incremental discrimination. These findings support the potential use of HbA1c as a pragmatic first-step triage tool to prioritize confirmatory OGTT in Vietnamese women with PMOS, while reinforcing that HbA1c should not replace OGTT for definitive glycemic classification (1).

Our finding that the Youden-derived HbA1c threshold for 2hPG-defined post-load dysglycemia was below 5.7% (39 mmol/mol) is consistent with the concern that diagnostic cutoffs derived from general populations may not perform optimally in younger Asian women with relatively low BMI and PMOS (4–6, 8). In the present analysis, the ADA HbA1c threshold of 5.7% (39 mmol/mol) had high specificity but low sensitivity for 2hPG-defined post-load dysglycemia, whereas the Youden-derived threshold of approximately 5.6% (38 mmol/mol) improved sensitivity but should be interpreted only as an exploratory statistical reference point, not as a clinically validated cutoff. Bootstrap resampling demonstrated variability in the selected cutoffs, particularly for the diabetes-range endpoint, supporting interpretation of approximately 5.6% as a threshold region rather than a reproducible biological boundary. This pattern suggests that the conventional threshold may under-detect post-load glucose abnormalities in this population. Similar observations have been reported in previous studies of women with PMOS, in which HbA1c showed only modest agreement with OGTT for detecting prediabetes or type 2 diabetes (11–13). Evidence from Asian populations also supports the plausibility of lower or population-specific HbA1c thresholds, with studies in Asian Indian, Chinese, and multi-ethnic Singaporean cohorts reporting clinically relevant variation in HbA1c performance across populations (15, 16, 24). Because a pre-OGTT triage strategy places greater weight on avoiding missed diabetes-range hyperglycemia than on reducing OGTT use, an exploratory point-estimate sensitivity target of 90% was selected for the diabetes-range endpoint during revision. The highest evaluated HbA1c cutoff meeting this criterion was 5.3% (34 mmol/mol), with 93.4% sensitivity and 34.5% specificity; however, 670 of every 1,000 women screened would still undergo an OGTT, underscoring the substantial testing burden.

The interpretation of HbA1c performance depends strongly on the reference standard. In this study, this was deliberately defined using only 2hPG during OGTT, rather than a composite definition including HbA1c or FPG. This approach avoided incorporation bias because HbA1c and FPG were the index tests under evaluation (25). Composite definitions that incorporate index-test information can inflate apparent diagnostic performance. For dysglycemia, HbA1c had higher apparent AUCs under both the first-visit glucose and OGTT-visit FPG composite definitions than under the 2hPG-only reference standard, consistent with incorporation bias (**Supplementary Table S5**). Future studies evaluating HbA1c in PMOS should therefore report non-circular estimates against post-load glucose-defined outcomes. Accordingly, a single 2hPG value ≥200 mg/dL in this study was treated as 2hPG-defined diabetes-range hyperglycemia rather than a confirmed diagnosis of diabetes; the 2026 ADA Standards require confirmatory testing when unequivocal hyperglycemia is absent (8).

The combined HbA1c + FPG model requires cautious interpretation. Within the fitted linear logistic models, FPG was positively associated with both endpoints after accounting for HbA1c. Tests of departure from linearity indicated non-linearity for both HbA1c and FPG at the post-load dysglycemia endpoint (**Supplementary Table S11**). The linear logistic model should therefore be regarded as an approximation, and its performance should not be interpreted as establishing the maximum incremental value obtainable from FPG. However, this conditional association translated into only small numerical increases in AUC: 0.010 for post-load dysglycemia and 0.022 for diabetes-range 2hPG hyperglycemia. The 95% CIs for both paired differences included zero, providing no statistically significant evidence that the combined model improved discrimination over HbA1c alone. An additional predictor may be associated with an outcome without materially improving the ranking of participants with and without that outcome. These results neither establish superiority of the combined model nor demonstrate equivalence between the models. Given the additional complexity and need for external validation, the combined model should not currently be presented as a superior screening approach.

The lower discriminatory performance of FPG for 2hPG-defined post-load dysglycemia is biologically plausible. In women with PMOS, early glucose abnormalities may present predominantly as post-load hyperglycemia, whereas fasting glucose can remain within the normal range. This explains why FPG alone may miss a substantial proportion of women with impaired glucose tolerance and why the OGTT remains the most accurate test for glycemic assessment in PMOS (1, 9, 10, 14). The prespecified probability cutoffs for the combined model also produced very small high-risk strata in the present analysis, making these categories unstable for clinical interpretation. These findings indicate that the original prespecified probability thresholds were not well calibrated for the non-circular 2hPG-defined reference standard and should not be used clinically without recalibration and external validation. Therefore, risk-group findings should be framed as exploratory and not as a validated risk-stratification algorithm. In the revised analysis, FPG was the OGTT-visit sample obtained after an overnight fast of at least 8 hours; the first-visit glucose sample obtained after a minimum 4-hour fast was retained only as a sensitivity analysis. Using the true fasting sample improved FPG discrimination compared with the first-visit measurement, although its AUC remained lower than that of HbA1c for both 2hPG-defined endpoints.

Clinically, these findings illustrate the potential and limitations of a selective testing strategy. Universal OGTT remains ideal, especially before fertility treatment or pregnancy planning. However, it may be difficult to implement consistently in busy or resource-limited settings. HbA1c may help prioritize OGTT, but the trade-off remains substantial: at 5.3% (34 mmol/mol), sensitivity for the diabetes-range endpoint was 93.4%, yet approximately 670 OGTTs would still be performed per 1,000 women screened. The Youden-derived thresholds should therefore remain exploratory, and a normal HbA1c result cannot reliably rule out 2hPG-defined post-load dysglycemia. Clinical judgment and additional risk factors, including age, central adiposity, prior abnormal glucose metabolism, family history, and planned pregnancy, should continue to guide the indication for OGTT.

The change in terminology from PCOS to PMOS is relevant to the present study because the metabolic component of the syndrome is central to both disease burden and clinical management. Our findings are consistent with this broader endocrine–metabolic framing: in this Vietnamese fertility-care cohort, in which 61.3% of women had BMI <23 kg/m², post-load glucose abnormalities were common, and conventional HbA1c thresholds showed limited sensitivity. These results support systematic metabolic assessment as an important component of care for women with PMOS, while emphasizing that diagnostic criteria remain unchanged.

This study has several strengths. It was based on a large prospective PMOS cohort with standardized glycemic assessment, complete HbA1c and FPG data in the analysis population, and a non-circular reference standard based only on 2hPG during OGTT. The analysis also included ROC analysis, Youden-optimal cutoffs, paired AUC comparisons, internal validation, calibration assessment, and decision-curve analysis, providing a rigorous evaluation of diagnostic performance. These methods strengthen the reliability of the finding that HbA1c was the best single index test in this cohort, although its discrimination for 2hPG-defined post-load dysglycemia was modest.

Several limitations should be acknowledged. First, participants were recruited from a single tertiary fertility center in Vietnam. Their BMI distribution, prevalence and severity of dysglycemia, and other clinical characteristics may differ from those of women with PMOS identified in community-based, primary-care, or non-fertility settings. Such differences in case mix may introduce spectrum bias and alter the observed AUCs, sensitivity, specificity, and predictive values at a fixed HbA1c threshold. Accordingly, the thresholds derived in this single cohort should be considered population-specific and exploratory. External validation in independent community-based and clinical PMOS cohorts with broader BMI and ethnic distributions is required before clinical implementation. Because the thresholds were derived and internally validated in the same cohort, the bootstrap results quantify internal stability but do not establish transportability or external validity. Second, OGTT was performed at a single time point, and short-term biological variability in glucose tolerance cannot be excluded. Third, the primary FPG measure was obtained at the OGTT visit after an overnight fast of at least 8 hours; the first-visit glucose measurement obtained after a minimum 4-hour fast was retained only as a sensitivity analysis and should not be interpreted as diagnostic FPG. Fourth, non-glycemic determinants of HbA1c, including anemia, hemoglobin variants, iron status, and altered erythrocyte turnover, were not fully assessed. Finally, because the primary reference standard was based only on 2hPG, the analysis primarily evaluated detection of post-load hyperglycemia and may not capture glycemic abnormalities defined solely by FPG or HbA1c. The combined-model risk groups were unstable in the present analysis and should not be used clinically without recalibration and external validation. The diagnostic-accuracy estimates apply to women who remained eligible for OGTT after the initial assessment and do not establish first-line screening performance in an unselected fertility-care population.

In conclusion, in Vietnamese women with PMOS seeking fertility care, HbA1c was the best-performing single test among those evaluated for detecting 2hPG-defined post-load dysglycemia and 2hPG-defined diabetes-range hyperglycemia, although its discrimination was modest. The Youden-derived HbA1c thresholds of approximately 5.6% (38 mmol/mol) should be considered exploratory statistical reference points rather than clinical cutoffs. A lower threshold of 5.3% (34 mmol/mol) improved sensitivity for detecting diabetes-range 2hPG. However, HbA1c should not be used to rule out 2hPG-defined post-load dysglycemia or replace OGTT for definitive glycemic classification. External validation is required before any HbA1c threshold can be recommended for routine clinical use.

## Data Availability

De-identified individual participant data will not be made publicly available or shared externally because of institutional data-governance restrictions and the terms of the original study registration. The analysis code will be available from the corresponding author upon reasonable request, subject to institutional approval.
