# Supplementary material for: Glycated hemoglobin thresholds for detecting 2-hour plasma glucose-defined abnormal glucose metabolism in Vietnamese women with polyendocrine metabolic ovarian syndrome
Source: Front Endocrinol (Lausanne). 2026 Sep 30;17:1968625. doi: 10.3389/fendo.2026.1968625 (PMC13661364; doi:10.3389/fendo.2026.1968625)
Supplement: Supplementary file 1 [file Supplementaryfile1.docx]

**Supplementary Material**

**Table S1** Standards for Reporting of Diagnostic Accuracy Studies (STARD)-style participant-flow log (counts at each gate per endpoint × model).

| **Step** | **Participants (n)** |
| --- | --- |
| Enrolled in the original prospective cohort | 1208 |
| Excluded: diabetes already diagnosed before the study visit | 8 |
| Assessed with HbA1c and first-visit plasma glucose after a protocol minimum fast of 4 hours | 1200 |
| Did not proceed to OGTT because first-visit HbA1c and/or ≥4-hour glucose met the original study’s protocol-defined diabetes-range exclusion criteria | 51 |
| Analysis cohort: underwent the 75-g OGTT with a 2hPG result | 1149 |
| HbA1c available (denominator for HbA1c-only models) | 1149 |
| FPG available (denominator for FPG-only models) | 1149 |
| HbA1c & FPG both available (denominator for combined model) | 1149 |
| Events: Post-load dysglycaemia (2hPG-defined, 2hPG ≥ 140 mg/dL) | 342 |
| Events: Diabetes-range hyperglycaemia (2hPG-defined, 2hPG ≥ 200 mg/dL) | 61 |
| Events: Composite-defined dysglycaemia (first-visit glucose) | 555 |
| Events: Composite-defined dysglycaemia (OGTT-visit FPG) | 495 |
| Events: Composite-defined diabetes-range hyperglycaemia | 61 |
| Recorded glucose-lowering treatment (metformin/insulin/GLP-1), assigned after glycaemic classification | 181 |

2hPG, 2-hour plasma glucose; ADA, American Diabetes Association; FPG, fasting plasma glucose; GLP-1, glucagon-like peptide-1; HbA1c, glycated haemoglobin; OGTT, oral glucose tolerance test.

**Table S2.** Diagnostic indices at all evaluated cut-offs (American Diabetes Association, Youden, fixed-specificity).

| **Endpoint** | **Test** | **Operating point** | **Cutoff** | **AUC** | **AUC_CI** | **Sens** | **Sens_CI** | **Spec** | **Spec_CI** | **PPV (95% CI)** | **NPV**  **(95% CI)** | **LR_pos (95% CI)** | **LR_neg (95% CI)** | **N** | **Events** |
| --- | --- | --- | --- | --- | --- | --- | --- | --- | --- | --- | --- | --- | --- | --- | --- |
| 2hPG ≥ 140 mg/dL | HbA1c | ADA 5.7% | 5.7 | 0.711 | (0.677-0.745) | 0.380 | (0.330-0.433) | 0.887 | (0.864-0.907) | 0.588 (0.522-0.651) | 0.772 (0.743-0.797) | 3.37 (2.66-4.27) | 0.70 (0.64-0.76) | 1149 | 342 |
| 2hPG ≥ 140 mg/dL | HbA1c | ADA 6.5% | 6.5 | 0.711 | (0.677-0.745) | 0.000 | (0.000-0.011) | 1.000 | (0.995-1.000) | NE | 0.702 (0.675-0.728) | NE | 1.00 (1.00-1.00) | 1149 | 342 |
| 2hPG ≥ 140 mg/dL | HbA1c | Youden | 5.58 | 0.711 | (0.677-0.745) | 0.538 | (0.485-0.590) | 0.805 | (0.777-0.831) | 0.540 (0.487-0.592) | 0.804 (0.776-0.830) | 2.77 (2.33-3.28) | 0.57 (0.51-0.65) | 1149 | 342 |
| 2hPG ≥ 140 mg/dL | HbA1c | Sensitivity >=90% | 5.13 | 0.711 | (0.677-0.745) | 0.901 | (0.864-0.928) | 0.202 | (0.176-0.231) | 0.324 (0.295-0.354) | 0.827 (0.769-0.874) | 1.13 (1.07-1.19) | 0.49 (0.35-0.70) | 1149 | 342 |
| 2hPG ≥ 140 mg/dL | FPG | ADA 5.6 mmol/L | 5.6 | 0.634 | (0.597-0.670) | 0.292 | (0.247-0.343) | 0.895 | (0.872-0.914) | 0.541 (0.469-0.611) | 0.749 (0.721-0.775) | 2.78 (2.14-3.60) | 0.79 (0.74-0.85) | 1149 | 342 |
| 2hPG ≥ 140 mg/dL | FPG | ADA 7.0 mmol/L | 7 | 0.634 | (0.597-0.670) | 0.006 | (0.002-0.021) | 1.000 | (0.995-1.000) | 1.000 (0.342-1.000) | 0.704 (0.677-0.729) | NE | 0.99 (0.99-1.00) | 1149 | 342 |
| 2hPG ≥ 140 mg/dL | FPG | Youden | 5.28 | 0.634 | (0.597-0.670) | 0.523 | (0.470-0.576) | 0.699 | (0.666-0.730) | 0.424 (0.378-0.472) | 0.776 (0.744-0.805) | 1.74 (1.50-2.01) | 0.68 (0.60-0.77) | 1149 | 342 |
| 2hPG ≥ 140 mg/dL | FPG | Sensitivity >=90% | 4.7 | 0.634 | (0.597-0.670) | 0.906 | (0.871-0.933) | 0.139 | (0.117-0.164) | 0.308 (0.281-0.338) | 0.778 (0.703-0.838) | 1.05 (1.01-1.10) | 0.67 (0.46-0.98) | 1149 | 342 |
| 2hPG ≥ 140 mg/dL | HbA1c + FPG | Youden | 0.313 | 0.721 | (0.687-0.755) | 0.623 | (0.570-0.673) | 0.736 | (0.705-0.765) | 0.500 (0.453-0.547) | 0.822 (0.792-0.848) | 2.36 (2.05-2.72) | 0.51 (0.44-0.59) | 1149 | 342 |
| 2hPG ≥ 140 mg/dL | HbA1c + FPG | Specificity >=0.90 | 0.449 | 0.721 | (0.687-0.755) | 0.365 | (0.316-0.418) | 0.901 | (0.878-0.920) | 0.610 (0.542-0.674) | 0.770 (0.742-0.796) | 3.69 (2.87-4.74) | 0.70 (0.65-0.77) | 1149 | 342 |
| 2hPG ≥ 200 mg/dL | HbA1c | ADA 5.7% | 5.7 | 0.799 | (0.739-0.858) | 0.590 | (0.465-0.705) | 0.830 | (0.806-0.851) | 0.163 (0.120-0.217) | 0.973 (0.961-0.982) | 3.47 (2.71-4.44) | 0.49 (0.36-0.67) | 1149 | 61 |
| 2hPG ≥ 200 mg/dL | HbA1c | ADA 6.5% | 6.5 | 0.799 | (0.739-0.858) | 0.000 | (0.000-0.059) | 1.000 | (0.996-1.000) | NE | 0.947 (0.932-0.958) | NE | 1.00 (1.00-1.00) | 1149 | 61 |
| 2hPG ≥ 200 mg/dL | HbA1c | Youden | 5.62 | 0.799 | (0.739-0.858) | 0.689 | (0.564-0.791) | 0.800 | (0.775-0.822) | 0.162 (0.122-0.211) | 0.979 (0.967-0.986) | 3.44 (2.80-4.22) | 0.39 (0.27-0.57) | 1149 | 61 |
| 2hPG ≥ 200 mg/dL | HbA1c | Sensitivity >=90% | 5.3 | 0.799 | (0.739-0.858) | 0.934 | (0.843-0.974) | 0.345 | (0.317-0.373) | 0.074 (0.058-0.095) | 0.989 (0.973-0.996) | 1.43 (1.32-1.54) | 0.19 (0.07-0.49) | 1149 | 61 |
| 2hPG ≥ 200 mg/dL | FPG | ADA 5.6 mmol/L | 5.6 | 0.719 | (0.650-0.788) | 0.443 | (0.325-0.567) | 0.855 | (0.833-0.874) | 0.146 (0.102-0.204) | 0.965 (0.951-0.975) | 3.05 (2.22-4.18) | 0.65 (0.52-0.82) | 1149 | 61 |
| 2hPG ≥ 200 mg/dL | FPG | ADA 7.0 mmol/L | 7 | 0.719 | (0.650-0.788) | 0.033 | (0.009-0.112) | 1.000 | (0.996-1.000) | 1.000 (0.342-1.000) | 0.949 (0.934-0.960) | NE | 0.97 (0.92-1.01) | 1149 | 61 |

Values are diagnostic performance estimates with 95% confidence interval values where applicable. Prespecified cutoffs refer to probability thresholds for the combined HbA1c+FPG model. 2hPG, 2-hour plasma glucose; AUC, area under the receiver operating characteristic curve; CI, confidence interval; FPG, fasting plasma glucose; HbA1c, glycated haemoglobin; LR+, positive likelihood ratio; LR−, negative likelihood ratio; NPV, negative predictive value; PPV, positive predictive value; Sens, sensitivity; Spec, specificity.

**Table S3.** Pairwise paired DeLong area under the curve contrasts (glycated hemoglobin [HbA1c] vs. fasting plasma glucose [FPG], HbA1c vs. combined, and FPG vs. combined) for both endpoints.

| **Endpoint** | **Contrast** | **AUC_a** | **AUC_b** | **Diff** | **p_DeLong** | **n** |
| --- | --- | --- | --- | --- | --- | --- |
| 2hPG ≥ 140 mg/dL | HbA1c vs FPG | 0.711 | 0.634 | +0.077 (+0.035 to +0.120) | <0.001 | 1149 |
| 2hPG ≥ 140 mg/dL | HbA1c vs HbA1c+FPG | 0.711 | 0.721 | -0.010 (-0.023 to +0.002) | 0.105 | 1149 |
| 2hPG ≥ 140 mg/dL | FPG vs HbA1c+FPG | 0.634 | 0.721 | -0.087 (-0.121 to -0.054) | <0.001 | 1149 |
| 2hPG ≥ 200 mg/dL | HbA1c vs FPG | 0.799 | 0.719 | +0.080 (+0.005 to +0.155) | 0.037 | 1149 |
| 2hPG ≥ 200 mg/dL | HbA1c vs HbA1c+FPG | 0.799 | 0.821 | -0.022 (-0.049 to +0.005) | 0.105 | 1149 |
| 2hPG ≥ 200 mg/dL | FPG vs HbA1c+FPG | 0.719 | 0.821 | -0.102 (-0.157 to -0.046) | <0.001 | 1149 |

p-values are two-sided paired DeLong tests. 2hPG, 2-hour plasma glucose; AUC, area under the receiver operating characteristic curve; AUC_a, AUC of the first test in the contrast; AUC_b, AUC of the second test; Diff, AUC_a minus AUC_b (positive values favor the first test); FPG, fasting plasma glucose; HbA1c, glycated haemoglobin.

**Table S4.** Internal-validation metrics for the combined glycated hemoglobin + fasting plasma glucose model and single-test area under the curve values (post-load dysglycaemia and diabetes-range hyperglycaemia).

| **Model / Endpoint** | **n** | **Events** | **AUC apparent** | **AUC optimism-corrected** | **Brier apparent** | **Brier optimism-corrected** | **Calibration slope (corrected)** | **Calibration intercept (corrected)** | **Bootstrap, n** |
| --- | --- | --- | --- | --- | --- | --- | --- | --- | --- |
| HbA1c + FPG 🡪 Post-load dysglycaemia | 1149 | 342 | 0.721 | 0.720 | 0.177 | 0.178 | 0.996 | -0.000 | 500 |
| HbA1c + FPG 🡪 Diabetes-range hyperglycaemia | 1149 | 61 | 0.821 | 0.816 | 0.044 | 0.045 | 0.981 | +0.002 | 500 |
| HbA1c 🡪 Post-load dysglycaemia | 1149 | 342 | 0.711 (0.677-0.745) | — | — | — | — | — | — |
| FPG 🡪 Post-load dysglycaemia | 1149 | 342 | 0.634 (0.597-0.670) | — | — | — | — | — | — |
| HbA1c 🡪 Diabetes-range hyperglycaemia | 1149 | 61 | 0.799 (0.739-0.858) | — | — | — | — | — | — |
| FPG 🡪 Diabetes-range hyperglycaemia | 1149 | 61 | 0.719 (0.650-0.788) | — | — | — | — | — | — |

AUC, area under the receiver operating characteristic curve; CI, confidence interval; FPG, fasting plasma glucose; HbA1c, glycated haemoglobin. Bootstrap optimism correction used 500 resamples (Harrell procedure). Brier score reflects mean squared prediction error (a lower Brier score represents better model performance).

**Table S5.** Sensitivity analyses using original-study and OGTT-visit FPG composite definitions (n=1,149).

| **Endpoint** | **Test** | **Operating**  **point** | **Cutoff** | **AUC** | **AUC_CI** | **Sens** | **Sens_CI** | **Spec** | **Spec_CI** | **PPV** | **NPV** | **LR+** | **LR-** | **n** | **Events** |
| --- | --- | --- | --- | --- | --- | --- | --- | --- | --- | --- | --- | --- | --- | --- | --- |
| Composite-defined dysglycaemia (first-visit glucose) | HbA1c | ADA threshold / 5.7% (39 mmol/mol) | 5.7 | 0.767 | (0.740-0.795) | 0.398 | (0.358-0.439) | 1.000 | (0.994-1.000) | 1.000 (0.983-1.000) | 0.640 (0.609-0.670) | NE | 0.60 (0.56-0.64) | 1149 | 555 |
| Composite-defined dysglycaemia (first-visit glucose) | HbA1c | ADA threshold / 6.5% (48 mmol/mol) | 6.5 | 0.767 | (0.740-0.795) | 0.000 | (0.000-0.007) | 1.000 | (0.994-1.000) | NE | 0.517 (0.488-0.546) | NE | 1.00 (1.00-1.00) | 1149 | 555 |
| Composite-defined dysglycaemia (first-visit glucose) | HbA1c | Youden / 5.58% (37 mmol/mol) | 5.58 | 0.767 | (0.740-0.795) | 0.521 | (0.479-0.562) | 0.912 | (0.887-0.933) | 0.848 (0.805-0.882) | 0.671 (0.638-0.702) | 5.95 (4.53-7.80) | 0.53 (0.48-0.57) | 1149 | 555 |
| Composite-defined dysglycaemia (first-visit glucose) | FPG | ADA threshold / 5.6 mmol/L (100 mg/dL) | 5.6 | 0.681 | (0.650-0.712) | 0.272 | (0.237-0.311) | 0.943 | (0.921-0.959) | 0.816 (0.754-0.865) | 0.581 (0.550-0.612) | 4.75 (3.34-6.77) | 0.77 (0.73-0.82) | 1149 | 555 |
| Composite-defined dysglycaemia (first-visit glucose) | FPG | ADA threshold / 7 mmol/L (126 mg/dL) | 7 | 0.681 | (0.650-0.712) | 0.004 | (0.001-0.013) | 1.000 | (0.994-1.000) | 1.000 (0.342-1.000) | 0.518 (0.489-0.547) | NE | 1.00 (0.99-1.00) | 1149 | 555 |
| Composite-defined dysglycaemia (first-visit glucose) | FPG | Youden / 5.28 mmol/L (95 mg/dL) | 5.28 | 0.681 | (0.650-0.712) | 0.510 | (0.468-0.551) | 0.766 | (0.730-0.798) | 0.671 (0.624-0.714) | 0.626 (0.590-0.660) | 2.18 (1.84-2.57) | 0.64 (0.58-0.70) | 1149 | 555 |
| Composite-defined diabetes-range hyperglycaemia | HbA1c | ADA threshold / 5.7% (39 mmol/mol) | 5.7 | 0.799 | (0.739-0.858) | 0.590 | (0.465-0.705) | 0.830 | (0.806-0.851) | 0.163 (0.120-0.217) | 0.973 (0.961-0.982) | 3.47 (2.71-4.44) | 0.49 (0.36-0.67) | 1149 | 61 |
| Composite-defined diabetes-range hyperglycaemia | HbA1c | ADA threshold / 6.5% (48 mmol/mol) | 6.5 | 0.799 | (0.739-0.858) | 0.000 | (0.000-0.059) | 1.000 | (0.996-1.000) | NE | 0.947 (0.932-0.958) | NE | 1.00 (1.00-1.00) | 1149 | 61 |
| Composite-defined diabetes-range hyperglycaemia | HbA1c | Youden / 5.62% (38 mmol/mol) | 5.62 | 0.799 | (0.739-0.858) | 0.689 | (0.564-0.791) | 0.800 | (0.775-0.822) | 0.162 (0.122-0.211) | 0.979 (0.967-0.986) | 3.44 (2.80-4.22) | 0.39 (0.27-0.57) | 1149 | 61 |
| Composite-defined diabetes-range hyperglycaemia | FPG | ADA threshold / 5.6 mmol/L (100 mg/dL) | 5.6 | 0.719 | (0.650-0.788) | 0.443 | (0.325-0.567) | 0.855 | (0.833-0.874) | 0.146 (0.102-0.204) | 0.965 (0.951-0.975) | 3.05 (2.22-4.18) | 0.65 (0.52-0.82) | 1149 | 61 |
| Composite-defined diabetes-range hyperglycaemia | FPG | ADA threshold / 7 mmol/L (126 mg/dL) | 7 | 0.719 | (0.650-0.788) | 0.033 | (0.009-0.112) | 1.000 | (0.996-1.000) | 1.000 (0.342-1.000) | 0.949 (0.934-0.960) | NE | 0.97 (0.92-1.01) | 1149 | 61 |
| Composite-defined diabetes-range hyperglycaemia | FPG | Youden / 5.5 mmol/L (99 mg/dL) | 5.5 | 0.719 | (0.650-0.788) | 0.541 | (0.417-0.660) | 0.792 | (0.767-0.815) | 0.127 (0.092-0.174) | 0.969 (0.955-0.978) | 2.60 (2.01-3.37) | 0.58 (0.44-0.76) | 1149 | 61 |
| Composite-defined dysglycaemia (OGTT-visit FPG) | HbA1c | ADA threshold / 5.7% (39 mmol/mol) | 5.7 | 0.800 | (0.773-0.828) | 0.446 | (0.403-0.491) | 1.000 | (0.994-1.000) | 1.000 (0.983-1.000) | 0.705 (0.675-0.733) | NE | 0.55 (0.51-0.60) | 1149 | 495 |
| Composite-defined dysglycaemia (OGTT-visit FPG) | HbA1c | ADA threshold / 6.5% (48 mmol/mol) | 6.5 | 0.800 | (0.773-0.828) | 0.000 | (0.000-0.008) | 1.000 | (0.994-1.000) | NE | 0.569 (0.540-0.598) | NE | 1.00 (1.00-1.00) | 1149 | 495 |
| Composite-defined dysglycaemia (OGTT-visit FPG) | HbA1c | Youden / 5.58% (37 mmol/mol) | 5.58 | 0.800 | (0.773-0.828) | 0.580 | (0.536-0.623) | 0.917 | (0.894-0.936) | 0.842 (0.799-0.877) | 0.743 (0.711-0.772) | 7.02 (5.38-9.16) | 0.46 (0.41-0.51) | 1149 | 495 |
| Composite-defined dysglycaemia (OGTT-visit FPG) | FPG | ADA threshold / 5.6 mmol/L (100 mg/dL) | 5.6 | 0.735 | (0.704-0.765) | 0.374 | (0.332-0.417) | 1.000 | (0.994-1.000) | 1.000 (0.980-1.000) | 0.678 (0.648-0.707) | NE | 0.63 (0.59-0.67) | 1149 | 495 |
| Composite-defined dysglycaemia (OGTT-visit FPG) | FPG | ADA threshold / 7 mmol/L (126 mg/dL) | 7 | 0.735 | (0.704-0.765) | 0.004 | (0.001-0.015) | 1.000 | (0.994-1.000) | 1.000 (0.342-1.000) | 0.570 (0.541-0.599) | NE | 1.00 (0.99-1.00) | 1149 | 495 |
| Composite-defined dysglycaemia (OGTT-visit FPG) | FPG | Youden / 5.57 mmol/L (100 mg/dL) | 5.57 | 0.735 | (0.704-0.765) | 0.386 | (0.344-0.429) | 0.994 | (0.984-0.998) | 0.979 (0.948-0.992) | 0.681 (0.651-0.710) | 63.09 (23.60-168.65) | 0.62 (0.58-0.66) | 1149 | 495 |

Composite dysglycaemia was defined as HbA1c ≥5.7%, glucose ≥5.6 mmol/L, or 2hPG ≥7.8 mmol/L; diabetes-range thresholds were ≥6.5%, ≥7.0 mmol/L, and ≥11.1 mmol/L, respectively. The glucose component used either first-visit glucose (≥4-hour fast) or OGTT-visit FPG (≥8-hour fast). FPG in the Test column always refers to the OGTT-visit sample. Both versions yielded identical diabetes-range classifications, presented once. These composite analyses incorporate index-test information and are not independent validation. CI, confidence interval; AUC, area under the ROC curve; FPG, fasting plasma glucose; HbA1c, glycated haemoglobin; 2hPG, 2-hour plasma glucose; OGTT, oral glucose tolerance test; PPV/NPV, positive/negative predictive value; LR+/LR−, positive/negative likelihood ratio; Sens/Spec, sensitivity/specificity; NE, undefined or non-finite estimate. Confidence intervals are 95%.

**Table S6.** Descriptive analysis excluding participants with a recorded post-classification glucose-lowering treatment assignment.

| **Endpoint** | **Test** | **n** | **Events** | **AUC** | **AUC_CI** |
| --- | --- | --- | --- | --- | --- |
| Post-load dysglycaemia | HbA1c | 968 | 173 | 0.659 | (0.611-0.708) |
| Post-load dysglycaemia | FPG | 968 | 173 | 0.575 | (0.525-0.626) |
| Post-load dysglycaemia | HbA1c + FPG | 968 | 173 | 0.661 | (0.613-0.709) |
| Diabetes-range hyperglycaemia | HbA1c | 968 | 10 | 0.792 | (0.641-0.944) |
| Diabetes-range hyperglycaemia | FPG | 968 | 10 | 0.640 | (0.421-0.858) |
| Diabetes-range hyperglycaemia | HbA1c + FPG | 968 | 10 | 0.824 | (0.672-0.977) |

AUC, area under the receiver operating characteristic curve; CI, confidence interval; FPG, fasting plasma glucose; HbA1c, glycated haemoglobin. Treatment records represented management assigned after complete glycaemic classification rather than medication use at enrolment. Because exclusion was based on a post-classification variable strongly associated with glycaemic status, these estimates are descriptive and should not be interpreted as an untreated-cohort robustness analysis.

**Table S7.** Baseline characteristics of the analysis cohort stratified by the primary 2-hour plasma glucose (2hPG) reference standard.

| **Variable** | **Overall** | **Normal 2hPG** | **Impaired glucose tolerance** | **Diabetes-range 2hPG** | **p-value** |
| --- | --- | --- | --- | --- | --- |
| n | 1149 | 807 | 281 | 61 |  |
| Age, years | 29.2 ± 3.7 | 29.0 ± 3.5 | 29.6 ± 4.0 | 30.3 ± 3.9 | 0.003 |
| BMI, kg/m² | 22.8 ± 3.8 | 22.1 ± 3.4 | 24.3 ± 4.1 | 25.0 ± 4.2 | <0.001 |
| Waist circumference, cm | 77.2 ± 9.5 | 75.7 ± 8.7 | 80.5 ± 10.2 | 83.1 ± 10.0 | <0.001 |
| Waist–hip ratio | 0.9 ± 0.1 | 0.8 ± 0.1 | 0.9 ± 0.1 | 0.9 ± 0.1 | <0.001 |
| Central obesity (waist ≥80 cm), n (%) | 418 (36.4) | 235 (29.1) | 141 (50.2) | 42 (68.9) | <0.001 |
| BMI ≥23 kg/m², n (%) | 445 (38.7) | 246 (30.5) | 158 (56.2) | 41 (67.2) | <0.001 |
| Hyperandrogenism (HA), n (%) | 563 (49.0) | 364 (45.1) | 155 (55.2) | 44 (72.1) | <0.001 |
| Polycystic ovarian morphology (PCOM), n (%) | 1099 (95.6) | 773 (95.8) | 266 (94.7) | 60 (98.4) | 0.412 |
| PCOS phenotype, n (%) |  |  |  |  | <0.001 |
| A | 468 (40.7) | 299 (37.1) | 129 (45.9) | 40 (65.6) |  |
| B | 50 (4.4) | 34 (4.2) | 15 (5.3) | 1 (1.6) |  |
| C | 45 (3.9) | 31 (3.8) | 11 (3.9) | 3 (4.9) |  |
| D | 586 (51.0) | 443 (54.9) | 126 (44.8) | 17 (27.9) |  |
| HbA1c, % | 5.4 ± 0.3 | 5.3 ± 0.3 | 5.5 ± 0.3 | 5.8 ± 0.3 | <0.001 |
| Fasting plasma glucose, mmol/L | 5.2 ± 0.5 | 5.1 ± 0.4 | 5.3 ± 0.5 | 5.6 ± 0.6 | <0.001 |
| 2-h plasma glucose, mmol/L | 7.2 ± 1.9 | 6.2 ± 0.9 | 9.1 ± 0.9 | 12.3 ± 1.1 | <0.001 |
| AMH, ng/mL | 9.1 ± 4.4 | 9.1 ± 4.4 | 9.2 ± 4.7 | 9.0 ± 3.8 | 0.906 |
| Total cholesterol, mmol/L | 5.2 ± 0.9 | 5.1 ± 0.9 | 5.3 ± 1.0 | 5.4 ± 0.9 | <0.001 |
| HDL cholesterol, mmol/L | 1.3 ± 0.3 | 1.3 ± 0.3 | 1.2 ± 0.4 | 1.2 ± 0.3 | <0.001 |
| LDL cholesterol, mmol/L | 3.4 ± 0.8 | 3.4 ± 0.8 | 3.6 ± 0.9 | 3.7 ± 0.9 | <0.001 |
| Triglycerides, mmol/L | 1.4 (1.0–2.2) | 1.3 (0.9–2.0) | 1.8 (1.3–2.8) | 2.1 (1.4–2.8) | <0.001 |
| Family history of diabetes, n (%) | 264 (23.0) | 157 (19.5) | 85 (30.2) | 22 (36.1) | <0.001 |

Continuous variables are mean ± SD or median (interquartile range); categorical variables are n (%). Groups are defined by 2hPG during a 75-g oral glucose tolerance test: normal 2hPG <140 mg/dL, impaired glucose tolerance 140 to <200 mg/dL, diabetes-range 2hPG ≥200 mg/dL. p values are from one-way ANOVA or Kruskal-Wallis for continuous variables and chi-squared or Fisher exact test for categorical variables. AMH, anti-Mullerian hormone; BMI, body mass index; HbA1c, glycated haemoglobin; HDL, high-density lipoprotein; HOMA-IR, homeostatic model assessment of insulin resistance; LDL, low-density lipoprotein; PCOM, polycystic ovarian morphology.

**Table S8.** Post-classification glucose-lowering treatment records by 2-hour plasma glucose category.

| **2hPG category** | **With a treatment record, n** | **Total, n** | **%** |
| --- | --- | --- | --- |
| Normal 2hPG | 12 | 807 | 1.5 |
| Impaired glucose tolerance | 118 | 281 | 42.0 |
| Diabetes-range 2hPG | 51 | 61 | 83.6 |

The treatment variables recorded management decisions made after complete glycaemic classification, not medication use at enrolment. Accordingly, this table describes post-classification management assignment rather than treatment exposure during the interval between the index tests and reference standard. 2hPG, 2-hour plasma glucose.

**Table S9.** Discrimination and paired contrasts using OGTT-visit FPG and the first-visit short-fast glucose measurement.

| **Glucose measurement** | **Endpoint** | **Test or contrast** | **n** | **Events** | **AUC or difference** | **95% CI** |
| --- | --- | --- | --- | --- | --- | --- |
| OGTT-visit fasting glucose (>=8 h fast) | Post-load dysglycaemia (2hPG >=7.8 mmol/L) | HbA1c | 1149 | 342 | 0.711 | (0.677 to 0.745) |
| OGTT-visit fasting glucose (>=8 h fast) | Post-load dysglycaemia (2hPG >=7.8 mmol/L) | FPG | 1149 | 342 | 0.634 | (0.597 to 0.670) |
| OGTT-visit fasting glucose (>=8 h fast) | Post-load dysglycaemia (2hPG >=7.8 mmol/L) | HbA1c + FPG | 1149 | 342 | 0.721 | (0.687 to 0.755) |
| OGTT-visit fasting glucose (>=8 h fast) | Post-load dysglycaemia (2hPG >=7.8 mmol/L) | HbA1c vs FPG | 1149 | 342 | +0.077 | (+0.035 to +0.120), p <0.001 |
| OGTT-visit fasting glucose (>=8 h fast) | Post-load dysglycaemia (2hPG >=7.8 mmol/L) | HbA1c vs HbA1c+FPG | 1149 | 342 | -0.010 | (-0.023 to +0.002), p = 0.105 |
| OGTT-visit fasting glucose (>=8 h fast) | Post-load dysglycaemia (2hPG >=7.8 mmol/L) | FPG vs HbA1c+FPG | 1149 | 342 | -0.087 | (-0.121 to -0.054), p <0.001 |
| OGTT-visit fasting glucose (>=8 h fast) | Diabetes-range 2hPG (>=11.1 mmol/L) | HbA1c | 1149 | 61 | 0.799 | (0.739 to 0.858) |
| OGTT-visit fasting glucose (>=8 h fast) | Diabetes-range 2hPG (>=11.1 mmol/L) | FPG | 1149 | 61 | 0.719 | (0.650 to 0.788) |
| OGTT-visit fasting glucose (>=8 h fast) | Diabetes-range 2hPG (>=11.1 mmol/L) | HbA1c + FPG | 1149 | 61 | 0.821 | (0.764 to 0.877) |
| OGTT-visit fasting glucose (>=8 h fast) | Diabetes-range 2hPG (>=11.1 mmol/L) | HbA1c vs FPG | 1149 | 61 | +0.080 | (+0.005 to +0.155), p = 0.037 |
| OGTT-visit fasting glucose (>=8 h fast) | Diabetes-range 2hPG (>=11.1 mmol/L) | HbA1c vs HbA1c+FPG | 1149 | 61 | -0.022 | (-0.049 to +0.005), p = 0.105 |
| OGTT-visit fasting glucose (>=8 h fast) | Diabetes-range 2hPG (>=11.1 mmol/L) | FPG vs HbA1c+FPG | 1149 | 61 | -0.102 | (-0.157 to -0.046), p <0.001 |
| First-visit glucose (>=4 h fast, as submitted) | Post-load dysglycaemia (2hPG >=7.8 mmol/L) | HbA1c | 1149 | 342 | 0.711 | (0.677 to 0.745) |
| First-visit glucose (>=4 h fast, as submitted) | Post-load dysglycaemia (2hPG >=7.8 mmol/L) | Short-fast glucose | 1149 | 342 | 0.572 | (0.535 to 0.610) |
| First-visit glucose (>=4 h fast, as submitted) | Post-load dysglycaemia (2hPG >=7.8 mmol/L) | HbA1c + Short-fast glucose | 1149 | 342 | 0.712 | (0.678 to 0.747) |
| First-visit glucose (>=4 h fast, as submitted) | Post-load dysglycaemia (2hPG >=7.8 mmol/L) | HbA1c vs Short-fast glucose | 1149 | 342 | +0.138 | (+0.093 to +0.184), p <0.001 |
| First-visit glucose (>=4 h fast, as submitted) | Post-load dysglycaemia (2hPG >=7.8 mmol/L) | HbA1c vs HbA1c + Short-fast glucose | 1149 | 342 | -0.002 | (-0.006 to +0.003), p = 0.523 |
| First-visit glucose (>=4 h fast, as submitted) | Post-load dysglycaemia (2hPG >=7.8 mmol/L) | Short-fast glucose vs HbA1c + Short-fast glucose | 1149 | 342 | -0.140 | (-0.182 to -0.098), p <0.001 |
| First-visit glucose (>=4 h fast, as submitted) | Diabetes-range 2hPG (>=11.1 mmol/L) | HbA1c | 1149 | 61 | 0.799 | (0.739 to 0.858) |
| First-visit glucose (>=4 h fast, as submitted) | Diabetes-range 2hPG (>=11.1 mmol/L) | Short-fast glucose | 1149 | 61 | 0.619 | (0.533 to 0.705) |
| First-visit glucose (>=4 h fast, as submitted) | Diabetes-range 2hPG (>=11.1 mmol/L) | HbA1c + Short-fast glucose | 1149 | 61 | 0.803 | (0.744 to 0.862) |
| First-visit glucose (>=4 h fast, as submitted) | Diabetes-range 2hPG (>=11.1 mmol/L) | HbA1c vs Short-fast glucose | 1149 | 61 | +0.180 | (+0.083 to +0.276), p <0.001 |
| First-visit glucose (>=4 h fast, as submitted) | Diabetes-range 2hPG (>=11.1 mmol/L) | HbA1c vs HbA1c + Short-fast glucose | 1149 | 61 | -0.004 | (-0.015 to +0.007), p = 0.485 |
| First-visit glucose (>=4 h fast, as submitted) | Diabetes-range 2hPG (>=11.1 mmol/L) | Short-fast glucose vs HbA1c + Short-fast glucose | 1149 | 61 | -0.184 | (-0.273 to -0.094), p <0.001 |

The OGTT-visit sample follows an overnight fast of at least 8 hours and is the index test in the primary analysis; the first-visit sample follows a minimum 4-hour fast and is the variable used in the submitted version. Only the OGTT-visit measurement meets the conventional definition of FPG; the first-visit ≥4-hour glucose measurement is presented solely as a sensitivity analysis. Contrast rows give the paired DeLong difference with its interval and p value. AUC, area under the receiver operating characteristic curve; CI, confidence interval; FPG, fasting plasma glucose; HbA1c, glycated haemoglobin; OGTT, oral glucose tolerance test.

**Table S10.** Full specification of the combined glycated haemoglobin + fasting plasma glucose model.

| **Fasting glucose variable** | **Endpoint** | **Term** | **Coefficient** | **SE** | **z** | **p** | **n** | **Events** |
| --- | --- | --- | --- | --- | --- | --- | --- | --- |
| OGTT-visit fasting glucose (>=8 h fast) | Post-load dysglycaemia (2hPG >=7.8 mmol/L) | intercept | -17.3331 | 1.4344 | -12.08 | <0.001 | 1149 | 342 |
| OGTT-visit fasting glucose (>=8 h fast) | Post-load dysglycaemia (2hPG >=7.8 mmol/L) | HbA1c (%) | +2.3973 | 0.2583 | +9.28 | <0.001 | 1149 | 342 |
| OGTT-visit fasting glucose (>=8 h fast) | Post-load dysglycaemia (2hPG >=7.8 mmol/L) | FPG (mmol/L) | +0.6499 | 0.1659 | +3.92 | <0.001 | 1149 | 342 |
| OGTT-visit fasting glucose (>=8 h fast) | Diabetes-range 2hPG (>=11.1 mmol/L) | intercept | -24.4753 | 2.5147 | -9.73 | <0.001 | 1149 | 61 |
| OGTT-visit fasting glucose (>=8 h fast) | Diabetes-range 2hPG (>=11.1 mmol/L) | HbA1c (%) | +2.8679 | 0.4755 | +6.03 | <0.001 | 1149 | 61 |
| OGTT-visit fasting glucose (>=8 h fast) | Diabetes-range 2hPG (>=11.1 mmol/L) | FPG (mmol/L) | +1.0474 | 0.2979 | +3.52 | <0.001 | 1149 | 61 |

Unpenalised maximum likelihood, lbfgs solver. HbA1c is in NGSP percent and fasting plasma glucose in mmol/L, both continuous and untransformed. Only the OGTT-visit measurement meets the conventional definition of FPG; the first-visit ≥4-hour glucose measurement is presented solely as a sensitivity analysis. SE, standard error.

**Table S11.** Test of departure from linearity for each index test.

| **Endpoint** | **Variable** | **Likelihood ratio statistic** | **df** | **p** |
| --- | --- | --- | --- | --- |
| Post-load dysglycaemia (2hPG >=7.8 mmol/L) | HbA1c | 11.3 | 1 | <0.001 |
| Post-load dysglycaemia (2hPG >=7.8 mmol/L) | FPG (OGTT visit) | 18.7 | 1 | <0.001 |
| Diabetes-range 2hPG (>=11.1 mmol/L) | HbA1c | 0.0 | 1 | 0.917 |
| Diabetes-range 2hPG (>=11.1 mmol/L) | FPG (OGTT visit) | 0.7 | 1 | 0.398 |

A restricted cubic spline with three knots at the 10th, 50th and 90th percentiles was compared with the linear term by likelihood ratio, which carries one degree of freedom. df, degrees of freedom.

**Table S12.** Internal validation of the cut-point selection procedure.

| **Endpoint** | **Apparent cut-point, %** | **Bootstrap median, %** | **2.5th to 97.5th percentile, %** | **Resamples selecting 5.5-5.7%** | **Sensitivity apparent** | **Sensitivity corrected** | **Specificity apparent** | **Specificity corrected** | **Resamples used** |
| --- | --- | --- | --- | --- | --- | --- | --- | --- | --- |
| Post-load dysglycaemia (2hPG >=7.8 mmol/L) | 5.58 | 5.56 | 5.41 to 5.58 | 81.6% | 0.538 | 0.524 | 0.805 | 0.799 | 1000 |
| Diabetes-range 2hPG (>=11.1 mmol/L) | 5.62 | 5.62 | 5.56 to 5.85 | 89.6% | 0.689 | 0.628 | 0.800 | 0.797 | 1000 |

In each resample the Youden-optimal HbA1c cut-point was reselected and applied to the observations left out of that resample. Optimism is the mean difference between in-sample and out-of-bag performance, and the corrected value is the apparent value minus that optimism. Seed 20260826.

**Table S13.** Absolute consequences of each candidate glycated haemoglobin threshold per 1,000 women screened.

| **HbA1c threshold, %** | **Sensitivity, post-load (%)** | **Specificity, post-load (%)** | **Sensitivity, diabetes-range (%)** | **Specificity, diabetes-range (%)** | **OGTTs performed** | **Post-load cases detected** | **Post-load cases missed** | **Diabetes-range cases detected** | **Diabetes-range cases missed** |
| --- | --- | --- | --- | --- | --- | --- | --- | --- | --- |
| 5.2 | 88.3 | 26.1 | 96.7 | 22.9 | 782 | 263 | 35 | 51 | 2 |
| 5.3 | 82.5 | 39.5 | 93.4 | 34.5 | 670 | 245 | 52 | 50 | 3 |
| 5.4 | 73.7 | 56.3 | 88.5 | 49.4 | 527 | 219 | 78 | 47 | 6 |
| 5.5 | 60.8 | 70.5 | 73.8 | 63.1 | 388 | 181 | 117 | 39 | 14 |
| 5.6 | 50.3 | 81.8 | 72.1 | 74.7 | 278 | 150 | 148 | 38 | 15 |
| 5.7 | 38.0 | 88.7 | 59.0 | 83.0 | 192 | 113 | 185 | 31 | 22 |

Candidate HbA1c thresholds from 5.2% to 5.7% were evaluated at 0.1-percentage-point increments. Estimates assume that women with HbA1c at or above the evaluated threshold undergo OGTT; “cases missed” are reference-positive women below that threshold. The 90% criterion refers to the observed sensitivity point estimate for the diabetes-range endpoint. Counts were rounded independently and may therefore not sum exactly to the rounded prevalence totals. At the prevalence of the analysis cohort: 298 women with post-load dysglycaemia and 53 with diabetes-range 2hPG per 1,000. A sensitivity of 90% for the diabetes-range endpoint is reached only at 5.3% and below. OGTT, oral glucose tolerance test.

**Table S14.** Decision-curve analysis interpreted as a testing strategy.

| **Endpoint** | **Threshold probability** | **OGTTs accepted per additional case detected** | **Net benefit, HbA1c alone** | **Net benefit, OGTT for everyone** | **Equivalent net reduction in OGTTs per 1,000** |
| --- | --- | --- | --- | --- | --- |
| Post-load dysglycaemia (2hPG >=7.8 mmol/L) | 0.02 | 49 | +0.2833 | +0.2833 | 0 |
| Post-load dysglycaemia (2hPG >=7.8 mmol/L) | 0.03 | 32 | +0.2760 | +0.2759 | 1 |
| Post-load dysglycaemia (2hPG >=7.8 mmol/L) | 0.04 | 24 | +0.2686 | +0.2684 | 4 |
| Post-load dysglycaemia (2hPG >=7.8 mmol/L) | 0.05 | 19 | +0.2611 | +0.2607 | 7 |
| Post-load dysglycaemia (2hPG >=7.8 mmol/L) | 0.06 | 16 | +0.2528 | +0.2528 | -1 |
| Post-load dysglycaemia (2hPG >=7.8 mmol/L) | 0.07 | 13 | +0.2437 | +0.2448 | -14 |
| Post-load dysglycaemia (2hPG >=7.8 mmol/L) | 0.08 | 12 | +0.2336 | +0.2366 | -34 |
| Post-load dysglycaemia (2hPG >=7.8 mmol/L) | 0.09 | 10 | +0.2258 | +0.2282 | -24 |
| Post-load dysglycaemia (2hPG >=7.8 mmol/L) | 0.10 | 9 | +0.2166 | +0.2196 | -27 |
| Post-load dysglycaemia (2hPG >=7.8 mmol/L) | 0.11 | 8 | +0.2064 | +0.2108 | -36 |
| Post-load dysglycaemia (2hPG >=7.8 mmol/L) | 0.12 | 7 | +0.1937 | +0.2019 | -60 |
| Post-load dysglycaemia (2hPG >=7.8 mmol/L) | 0.13 | 7 | +0.1856 | +0.1927 | -47 |
| Post-load dysglycaemia (2hPG >=7.8 mmol/L) | 0.14 | 6 | +0.1770 | +0.1833 | -39 |
| Post-load dysglycaemia (2hPG >=7.8 mmol/L) | 0.15 | 6 | +0.1687 | +0.1737 | -28 |
| Post-load dysglycaemia (2hPG >=7.8 mmol/L) | 0.16 | 5 | +0.1629 | +0.1639 | -5 |
| Post-load dysglycaemia (2hPG >=7.8 mmol/L) | 0.17 | 5 | +0.1566 | +0.1538 | 14 |
| Post-load dysglycaemia (2hPG >=7.8 mmol/L) | 0.18 | 5 | +0.1535 | +0.1435 | 45 |
| Post-load dysglycaemia (2hPG >=7.8 mmol/L) | 0.19 | 4 | +0.1479 | +0.1329 | 64 |
| Post-load dysglycaemia (2hPG >=7.8 mmol/L) | 0.20 | 4 | +0.1416 | +0.1221 | 78 |
| Diabetes-range 2hPG (>=11.1 mmol/L) | 0.02 | 49 | +0.0355 | +0.0338 | 87 |
| Diabetes-range 2hPG (>=11.1 mmol/L) | 0.03 | 32 | +0.0305 | +0.0238 | 216 |
| Diabetes-range 2hPG (>=11.1 mmol/L) | 0.04 | 24 | +0.0246 | +0.0136 | 264 |
| Diabetes-range 2hPG (>=11.1 mmol/L) | 0.05 | 19 | +0.0246 | +0.0033 | 405 |
| Diabetes-range 2hPG (>=11.1 mmol/L) | 0.06 | 16 | +0.0244 | -0.0074 | 498 |
| Diabetes-range 2hPG (>=11.1 mmol/L) | 0.07 | 13 | +0.0211 | -0.0182 | 522 |
| Diabetes-range 2hPG (>=11.1 mmol/L) | 0.08 | 12 | +0.0172 | -0.0293 | 534 |
| Diabetes-range 2hPG (>=11.1 mmol/L) | 0.09 | 10 | +0.0159 | -0.0406 | 571 |
| Diabetes-range 2hPG (>=11.1 mmol/L) | 0.10 | 9 | +0.0156 | -0.0521 | 609 |
| Diabetes-range 2hPG (>=11.1 mmol/L) | 0.11 | 8 | +0.0143 | -0.0639 | 633 |
| Diabetes-range 2hPG (>=11.1 mmol/L) | 0.12 | 7 | +0.0165 | -0.0760 | 679 |
| Diabetes-range 2hPG (>=11.1 mmol/L) | 0.13 | 7 | +0.0152 | -0.0884 | 694 |
| Diabetes-range 2hPG (>=11.1 mmol/L) | 0.14 | 6 | +0.0152 | -0.1011 | 714 |
| Diabetes-range 2hPG (>=11.1 mmol/L) | 0.15 | 6 | +0.0139 | -0.1140 | 725 |
| Diabetes-range 2hPG (>=11.1 mmol/L) | 0.16 | 5 | +0.0139 | -0.1273 | 741 |
| Diabetes-range 2hPG (>=11.1 mmol/L) | 0.17 | 5 | +0.0110 | -0.1409 | 741 |
| Diabetes-range 2hPG (>=11.1 mmol/L) | 0.18 | 5 | +0.0106 | -0.1548 | 754 |
| Diabetes-range 2hPG (>=11.1 mmol/L) | 0.19 | 4 | +0.0105 | -0.1690 | 765 |
| Diabetes-range 2hPG (>=11.1 mmol/L) | 0.20 | 4 | +0.0100 | -0.1836 | 775 |

The action following a positive result is one 75-g OGTT and not the start of treatment. A threshold probability of 0.10 means accepting nine additional tests to detect one further case. OGTT, oral glucose tolerance test.

**Table S15.** Bounding analysis for the 51 participants with index-test results in whom the reference standard was not applied.

| **Endpoint** | **All 51 assumed reference-negative** | **Complete case**  **(n=1,149)** | **All 51 assumed reference-positive** | **n unverified** |
| --- | --- | --- | --- | --- |
| Post-load dysglycaemia (2hPG >=7.8 mmol/L) | 0.672 (0.638 to 0.706) | 0.711 (0.677 to 0.745) | 0.744 (0.712 to 0.775) | 51 |
| Diabetes-range 2hPG (>=11.1 mmol/L) | 0.767 (0.709 to 0.825) | 0.799 (0.739 to 0.858) | 0.870 (0.830 to 0.910) | 51 |

Values are the area under the receiver operating characteristic curve for HbA1c with its 95% confidence interval. The reference-negative bound is the less plausible of the two, because 40 of the 51 unverified participants had HbA1c at or above 6.5%.

**Table S16.** Precision of the sensitivity estimate at each endpoint.

| **Endpoint** | **Events (reference-positive)** | **Sensitivity at the selected cut-point** | **Wilson 95% CI** | **Half-width, percentage points** | **Events needed for a half-width of 5 points** |
| --- | --- | --- | --- | --- | --- |
| Post-load dysglycaemia (2hPG >=7.8 mmol/L) | 342 | 0.538 | (0.485 to 0.590) | 5.3 | 382 |
| Diabetes-range 2hPG (>=11.1 mmol/L) | 61 | 0.689 | (0.564 to 0.791) | 11.3 | 330 |

Sample size is justified by the precision of the sensitivity estimate rather than by a hypothesis test (Flahault A, Cadilhac M, Thomas G. J Clin Epidemiol 2005;58:859-62). The diabetes-range endpoint is powered for estimation and not for threshold selection.

**Figure legends**

**Figure S1.** Apparent calibration of the combined HbA1c + FPG model for post-load dysglycaemia.

The model was fitted and evaluated in the same cohort of 1,149 women. Points show observed event proportions against mean predicted probabilities within deciles of predicted risk. The LOWESS curve was generated with frac=0.5 and no residual-based robustifying iterations (it=0). The plot is not corrected for optimism. FPG, fasting plasma glucose; HbA1c, glycated haemoglobin; LOWESS, locally weighted scatterplot smoothing.

**Figure S2.** Apparent calibration of the combined HbA1c + FPG model for diabetes-range hyperglycaemia.

The model was fitted and evaluated in the same cohort of 1,149 women. Points show observed event proportions against mean predicted probabilities within deciles of predicted risk. The LOWESS curve was generated with frac=0.5 and no residual-based robustifying iterations (it=0). The plot is not corrected for optimism; estimates at higher predicted probabilities are supported by few observations and should be interpreted cautiously. FPG, fasting plasma glucose; HbA1c, glycated haemoglobin; LOWESS, locally weighted scatterplot smoothing.

**Figure S3.** Subgroup area under the receiver operating characteristic curve forest plot - combined glycated hemoglobin + fasting plasma glucose, post-load dysglycaemia endpoint.

AUC, area under the receiver operating characteristic curve; BMI, body mass index (kg/m²); CI, confidence interval; FPG, fasting plasma glucose; HbA1c, glycated haemoglobin. Polyendocrine metabolic ovarian syndrome phenotypes: A, hyperandrogenism + ovulatory dysfunction + polycystic ovarian morphology; B, hyperandrogenism + ovulatory dysfunction; C, hyperandrogenism + polycystic ovarian morphology; D, ovulatory dysfunction + polycystic ovarian morphology.
